# Single cell sequencing analysis of respiratory syncytial virus–infected pediatric and adult human nose organoids reveals age differences, proliferative diversity and identifies novel cellular tropism

**DOI:** 10.1016/j.jinf.2025.106617

**Published:** 2025-09-18

**Authors:** Anubama Rajan, Divya Nagaraj, Carolyn Bomidi, Gina M. Aloisio, Ashley M. Murray, Emily M. Schultz, Amal Kambal, Mary K. Estes, Erin Nicholson, Vasanthi Avadhanula, Sarah E. Blutt, Pedro A. Piedra

**Affiliations:** a Department of Molecular Virology and Microbiology, Baylor College of Medicine, Houston, TX, USA; b Department of Pediatrics, Baylor College of Medicine, Houston, TX, USA

**Keywords:** Human nose organoid, RSV, Adult and Pediatric HNO, Single cell RNA seq

## Abstract

Respiratory syncytial virus (RSV) is a leading cause of infant death across the globe. Age is a significant factor that contributes to the severity of infection in young children. RSV primarily infects the ciliated cells of the airway epithelium, induces mucus hypersecretion, and impaired mucociliary clearance. Better understanding of RSV infection at the cellular level is needed for the development of effective therapeutic interventions. To investigate the age difference and comprehensively understand gene signatures associated with RSV infection, we performed single-cell transcriptomic analysis of adult and pediatric human nose organoids (HNOs) infected with RSV. Our analysis revealed a significant difference in transcriptomic signature associated with cellular differentiation and proliferative pathways between the adult and pediatric HNOs. Moreover, we found a distinct innate immune response to RSV infection, with pediatric HNO revealing a lower and dysregulated response. Through sub-clustering analysis of the ciliated cell population, we identified the primary ciliary cell as a novel and prominent susceptible ciliary cell type to RSV infection. Intriguingly and unexpectedly, we found that in the pediatric more than in the adult, HNO RSV infects other novel airway cells, including basal cells, and ionocytes/tuft cells, as demonstrated by increased RSV-gene counts and induction of interferon-related pathways. Together, our study provides the first HNO cell atlas dissecting the heterogeneity of RSV infection in airway epithelium between adult versus pediatric HNOs and identifies novel cell types that are susceptible to RSV infection, which altogether provides a key resource for research on RSV pathogenesis, therapeutics and vaccines.

## Introduction

Respiratory syncytial virus (RSV) is an important and ubiquitous viral pathogen that causes annual winter outbreaks in temperate climates and infects individuals across all age group.^1^ Globally, RSV is the major leading cause of lower respiratory infections (LRTI) in infants and young children and causes one in 50 deaths in children aged 0–60 months and one in every 28 deaths in infants less than 6 months of age.^2,3^ Symptoms and severity of RSV infection vary according to the age group.^4^ While healthy adults tend to show mild to moderate cold-like symptoms, young children, older adults, and immunocompromised individuals are more prone to pneumonia and severe LRTI, which at times results in death.^5,6^ There are multiple hypotheses associated with RSV disease severity including smaller distal airways and under-developed immune system of infants, reduced immune response in immunocompromised individuals, and immune senescence in older adults.^5,6^ Despite the known disease burden and RSV association with severe respiratory infections, there is a significant gap in our mechanistic understanding of age differences in susceptibility to RSV infection, especially regarding disease severity, virus-cell tropism, host-immune interactions, and therapeutic interventions. Especially, age-driven differences in RSV pathogenesis in the context of cell types in airway epithelium, changes in the epithelial cell populations in response to infection, and molecular mechanisms of RSV infection are still unidentified. This lack of knowledge is driven by the challenges associated with mimicking the multicellular complexity of the human airway epithelium. We believe addressing these questions requires a physiologically relevant ex vivo human model to dissect the heterogeneity of host–pathogen interactions in a human airway epithelium containing a broad array of different epithelial cell types.

Human airway epithelium is a heterogeneous, complex pseudostratified layer comprising of basal, goblet, club, and ciliated cells, together with rare cell population including ionocytes and tuft cells.^7^ For several years, the central dogma of RSV cell tropism has been exclusively restricted to the multi-ciliated cells of airway epithelium.^8,9^ The attachment (G) and fusion (F) glycoproteins are the major surface glycoproteins of RSV. The G-protein of RSV is predicted to target the ciliary cells - CX_3_C-chemokine receptor 1 (CX_3_CR1) for viral attachment,^10^ and the F protein uses cell receptors like insulin growth factor receptor 1 (IGFR-1)^11^ and nucleolin for virus entry,^12^ yet it is unclear how RSV RNA is found in other airway epithelial cells and extrapulmonary sites.^13,14^ Furthermore, using mechanical and chemical injury studies and in-vitro model *Persson* et al. explored the tropism of RSV in basal cells.^15^ Multiple studies show evidence that assault on airway basal cells causes dysregulation of airway epithelial homeostasis, abnormal cellular proliferation leading to aberrant expansion, and eventually to chronic inflammatory injury and severe respiratory diseases.^16–18^ Hence, understanding RSV cell tropism is an important determinant of severe disease progression, and a key unresolved question is whether there are alternate routes of infection or cell tropism for RSV, especially after induction of antiviral response by ciliary cells and downregulation of viral receptors like CX_3_CR1. This is also critical for the development of alternative therapeutic strategies targeting host factors when the virus develops resistance to monoclonal antibodies or when the vaccine is not efficacious or not available for the major risk groups.

A hallmark feature for RSV infection is hypersecretion of mucus and goblet cell hyperplasia^15,19–21^ and in particular, this phenotype is even more pronounced in infants and young children than adults.^22,23^ Nevertheless, it is not clear if this increased mucus secretion occurs due to increased upregulation of mucus secretion from existing goblet cells or expansion of goblet cells from basal cells or other airway cell types, such as club cells and ciliated cells. In addition, the exact molecular mechanism by which this mucus up-regulation occurs is not precisely known. Even more, no studies have shown evidence for direct infection of goblet cells with RSV or tracked the differentiation of other airway cell types to secretory goblet cells. Identifying these molecular pathways is critical for the development of effective anti-mucolytic compounds for combating RSV symptoms and mitigating airway obstruction from mucous plugs that are associated with severe RSV.

Altogether, the main objectives of this study are to generate an atlas of the adult and infant human nose cell populations and to identify how susceptibility to RSV infection changes with age, molecular aspects of RSV infection in infants and adults, and identify if there are novel cell types associated with RSV tropism. Towards this, we used the single-cell RNA sequencing (sc-RNA seq) method to establish a framework to investigate individual cell-type-specific RSV pathogenesis in an airway tissue-like environment, human nose organoids (HNOs) model.^24,25^ Our study integrates the use of differential gene expression analysis, flow cytometry and immunohistochemistry imaging of RSV-infected HNOs derived from both adults and pediatric subjects to determine the cell-type and age-type specific differences in adult and pediatric HNOs. We first created a sc-RNA-seq reference dataset, the atlas of the human adult and pediatric nose cell populations and used this framework to identify cellular populations infected by RSV and characterized the differences in RSV infection between adult and pediatric HNOs. Our study revealed a distinct alteration in proliferation pattern between adult and pediatric HNOs, a novel cell tropism of RSV to primary ciliated cells, as well as evidence for an unexpected tropism to airway basal cells and ionocytes, interestingly, only in the pediatric HNOs.

## Results

### Sc-RNA Seq of adult and pediatric HNOs shows distinct and similar nasal epithelial cell subpopulations

**Fig. 1.**
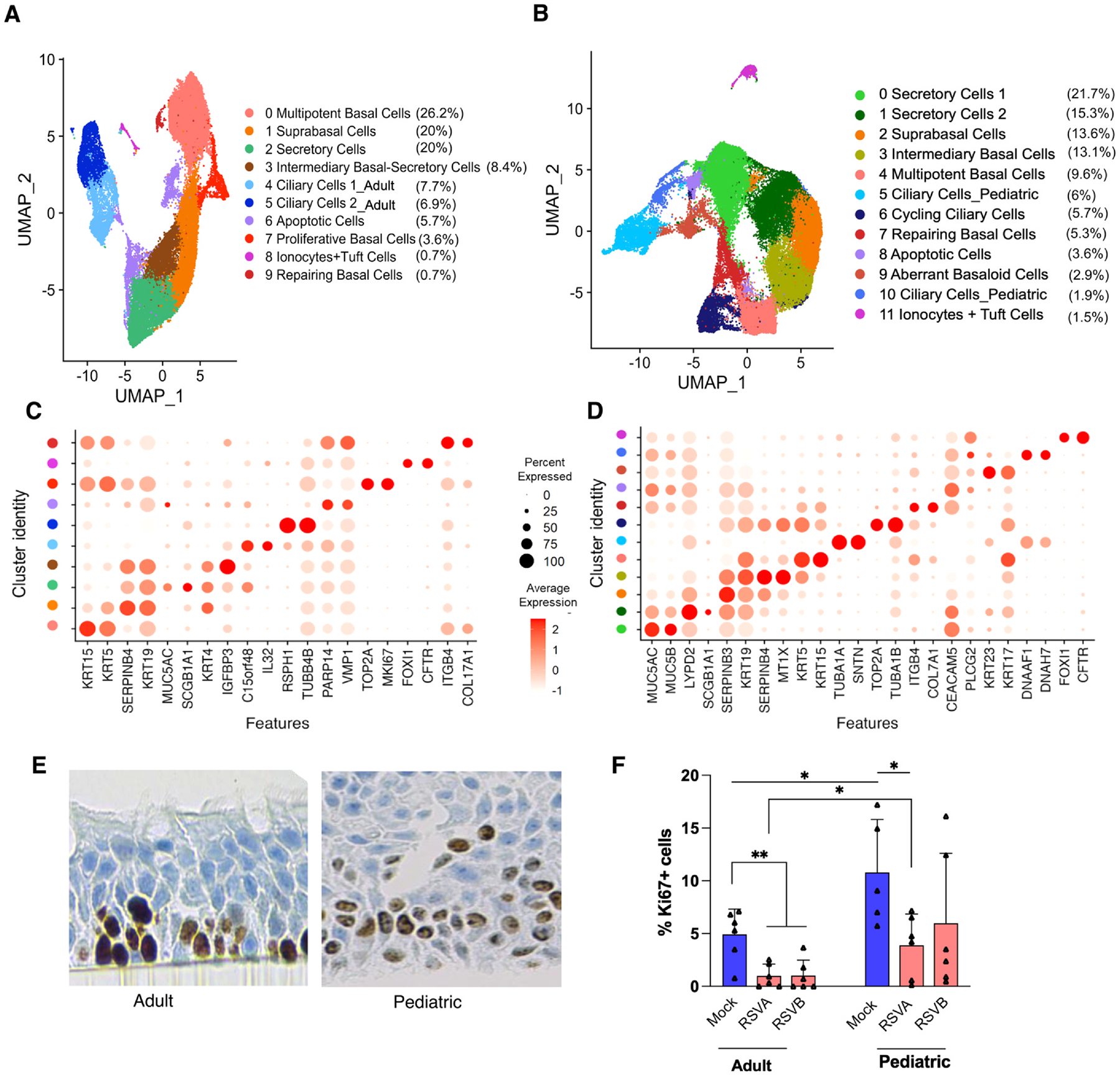
Unsupervised Clustering of Adult and Pediatric HNOs Reveals Age-Specific Basal and Ciliary Cell Subpopulations: (A) UMAP visualization of 35,818 respiratory epithelial cells from uninfected and RSV-infected adult HNOs. (B) UMAP visualization of 48,437 respiratory epithelial cells from uninfected and RSV-infected pediatric HNOs. The percentage proportion of individual cell types is included. (C, D) Dot plots showing marker genes used to define cell clusters, with dot size representing the percentage of cells expressing each marker and color intensity (salmon to red) indicating mean expression levels. (E) Immunofluorescent staining of KI67+ proliferating cells demonstrates differences in cellular proliferation between adult and pediatric HNOs. (F) Ki67-positive cells were quantitated for each group, and a student’s unpaired t-test was performed for differences between groups. *p < 0.05, **p < 0.01. Bars represent the mean of 3 replicates from 3 different adult and pediatric HNOs; error bars represent SD.

To dissect the primary differences in the nasal epithelium of an adult and a pediatric subject, epithelial cells from an adult HNO and a pediatric HNO were isolated and subjected to sc-RNA seq. Unsupervised clustering of a total of 84,255 cells identified 10 epithelial subpopulations in the adult HNO and 12 epithelial sub-populations in the pediatric HNO (Fig. 1A and B). Gene markers specific to each cluster were used to annotate and define cluster identities (Fig. 1C and D; Supplementary Tables S1 and S2). These subpopulations in the nasal epithelium included the majority of previously defined epithelial cell types of the airway epithelium, including ciliated cells, basal cells, secretory cells, and even the recently discovered ionocytes and tuft cells.^26,27^

In the adult HNO, we identified 3 different populations of basal cells: Multipotent Basal Cells (cluster 0, KRT5+ and KRT15+), suprabasal cells (cluster 1, SERPINB4+, KRT19+), and proliferative basal cells (cluster 7, KRT5+, MKI67+ and TOP2A+), each demonstrating a unique transcriptional signature. In the pediatric HNOs, in addition to the multipotent basal cells and suprabasal cells, we identified a unique cluster of basal cell population, aberrant basaloid cells, that are transcriptionally distinct from other basal cells and express KRT23 and KRT17 (cluster 9). These ABC were unique to the pediatric HNOs and express epithelial and mesenchymal markers (CDHR3, CDHR4) (Supplemental Data S2) and developmental transcription factors (HYDIN, DLEC1) (Supplemental Data S2) denoting an epithelial mesenchymal transition state and a development state of the cells.^27^ There are two distinct clusters of ciliary cells in the adult HNOs, with marked differences in the expression of genes related to maturity of cilium. The genes TUBA1A, TUBB4B, RSPH1 were enriched in the cluster Ciliary Cells 2 (cluster 5) and the genes GDF15, C15orf18, DNAAF1 more enriched in the cluster Ciliary Cells 1 (cluster 4) marking the different gene expression signatures related to a beating ciliary cell population versus an early deuterosomal cell population.^28^ In pediatric HNOs, surprisingly, we noticed a clear segregation of a third ciliary cell cluster, Cycling Ciliary Cells (cluster 6), marked by their proliferative ability (TOP2A+) in addition to Ciliary cells 1 (cluster 5; TUBA1A+ and SNTN+) and Ciliary Cells 2 (cluster 10; DNAH7 and DNAAF1), like adult HNOs. Adult HNOs had a single cluster of secretory cells (cluster 2) marked by the presence of MUC5AC and SCGB1A1, whereas; the pediatric HNOs had two distinct and larger populations of secretory cells potentially goblet cells (cluster 0, Secretory Cell 1, MUC5AC+ and MUC5B+) and club cells (cluster 1, Secretory Cells 2, LYPD2+ and SCGB1A1+) but with overlapping gene expression profile. Both adult and pediatric HNOs showed the presence of ionocytes-tuft cell clusters (adult HNO: cluster 8 and pediatric HNO: cluster 11) marked by the expression of FOXI1 and CFTR genes. We represented these populations as a single cluster in both datasets due to low abundances. In the adult HNO dataset, particularly, the limited cell numbers precluded robust separation into two distinct populations during the clustering analysis, as algorithms tend to merge rare transcriptionally related cell types.

Further, pediatric HNOs were more proliferative in nature and segregated into more clusters; we validated this through quantification of Ki67 proliferative marker. Uninfected pediatric HNOs had significantly more proliferating cells than uninfected adult HNOs (Fig. 1E and F), and RSV infection significantly decreased the number of Ki67+ cells compared to mock in both adults and pediatric HNOs. Interestingly, this reduction was more pronounced in adult HNOs as compared to pediatric HNOs (Fig. 1F).

### RSV-infected HNOs show variation in airway epithelial cell numbers, levels of viral transcripts and virus host receptor expression

**Fig. 2.**
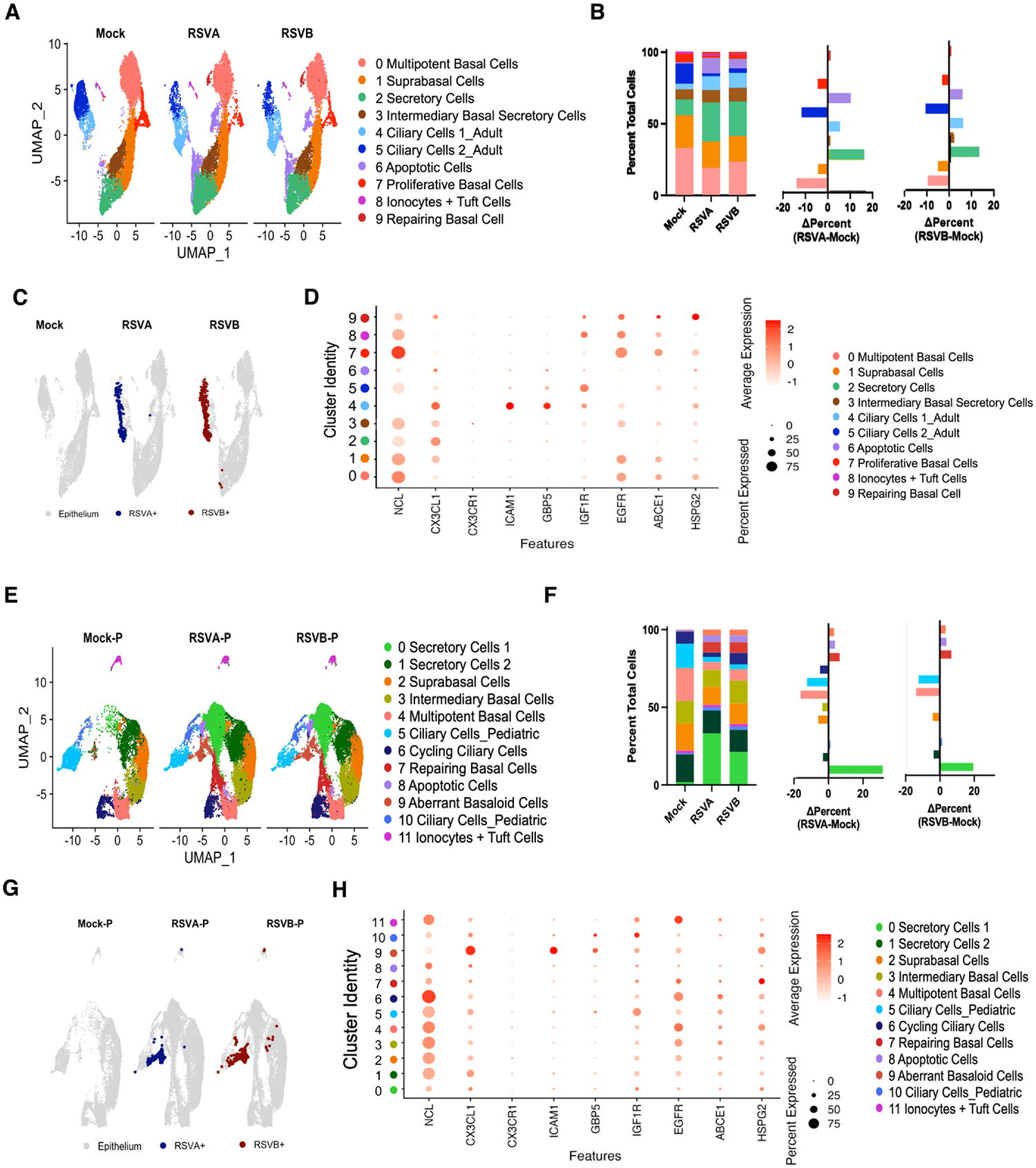
RSV-Infected HNOs Show Changes in Epithelial Cell Composition, Viral Gene Expression, and Host Receptor Profiles: (A) UMAPs of 13,964 respiratory epithelial cells from uninfected adult HNOs versus 11,579 cells from RSV/A infected adult HNOs and 10,275 cells from RSV/B infected adult HNOs. (B) Quantification of percent cells per cluster (Left) and difference in percent (infected–uninfected) (Right), ΔPercent indicates the difference in percent (infected–uninfected). (C) Distribution UMAP of mature airway epithelial cells containing RSV genes (NS1, NS2, N, and P). (D) Selected up-regulated hypothesized RSV receptor genes. (E) UMAPs of 11,128 respiratory epithelial cells from uninfected pediatric HNOs versus 19,985 cells from RSV/A infected pediatric HNOs and 17,324 cells from RSV/B infected pediatric HNOs. (F) Quantification of percent cells per cluster (Left) and difference in percent (infected–uninfected) (Right), ΔPercent indicates difference in percent (infected−uninfected). (G) Distribution UMAP of mature airway epithelial cells containing RSV genes (NS1, NS2, N, and P). (H) Selected up-regulated hypothesized RSV receptor genes.

Next, to characterize the cellular response of HNOs to RSV infection, we performed scRNA-seq 5 days post-infection using two contemporaneous RSV genotypes: RSV/A/ON and RSV/B/BA. Our aim was to delineate cell-type-specific responses within both adult and pediatric HNOs. Adult and pediatric HNOs infected with either RSV genotype had an additional cell cluster, Repairing Basal Cells, with markedly enriched and higher expression of ITGB4 and COL17A1 (Cluster 9 in adult HNOs; Cluster 7 in pediatric HNOs; Fig. 1C and D), involved in integrin and collagen XVII production for intercellular adhesion and anchoring to the basement membrane.^29^ Comparative analysis of uninfected versus RSV-infected HNOs revealed pronounced shifts across major epithelial lineages, specifically in ciliary, basal, and secretory cell clusters. Notably, both adult and pediatric models exhibited a substantial decrease in multipotent basal cells, ciliary cells (TUBA1A+ and TUBB4B+), along with a relative reduction in suprabasal cells. In contrast, secretory cell populations expanded significantly in response to infection (Fig. 2B and F). Furthermore, we observed a marked increase in apoptotic cell clusters (Cluster 6 in adult HNOs; Cluster 8 in pediatric HNOs), indicating damage and destruction of the nasal epithelium in response to RSV infection by either genotype (Fig. 2B and F).

Next, to investigate the cellular tropism of RSV at single-cell resolution, we identified cells expressing at least four key RSV genes (NS1, NS2, N, and P) indicative of active infection and viral replication^30^ and classified these as RSV + cells. In adult HNOs, RSV transcripts were predominantly restricted to the ciliary cell clusters (Cluster 4 and 5) (Fig. 2C). To our surprise, pediatric HNOs exhibited a broader viral tropism: RSV transcripts were detected not only in ciliary cells 1 and 2 (cluster 5 & cluster 10), but also in ABCs (cluster 9) and ionocytes/tuft cells (cluster 11) suggesting an expanded cellular susceptibility to RSV unique to the pediatric nasal epithelium (Fig. 2G).

To further explore host determinants of RSV susceptibility, we analyzed the expression of RSV receptors and infection-associated genes.^31^ The G protein of RSV can potentially attach to CX3C chemokine receptor 1 (CX3CR1) and heparan sulfated proteoglycans (HSPG) located on epithelial cells and require the interaction of the F protein for virus entry by binding to one or more of its cellular receptors; nucleolin (NCL), insulin-like growth factor-1 receptor (IGF1R), epidermal growth factor (EGFR), and intercellular adhesion molecule 1 (ICAM 1).^31^ Cell surface NCL is important for virus infection^12^ and in the adult HNO it was over expressed in the basal cell populations (clusters 0, 1 and 7) (Fig. 2D). In contrast, in the pediatric HNO, NCL was most highly expressed in cycling ciliary cells followed by several basal cell populations (clusters 2, 3 and 4) and ionocytes + tufts cells (cluster 11). *ICAM1* previously implicated in facilitating RSV entry and replication^32,33^ was significantly upregulated in infected HNOs. In adult RSV-infected HNOs, *ICAM1* expression was primarily localized to ciliary cell cluster (Clusters 4 and 5), while in pediatric HNOs, upregulated and higher expression was observed in both Ciliary Cells 1 (Cluster 5) and aberrant basal cells (Cluster 9) (Fig. 2C and G; Supplementary Figure 2A and B). This restricted up-regulation further points evidence towards cell tropism of RSV (Fig. 2C and G, and Supplementary Figure 2A and B).

Consistently, we noticed an increased expression of Guanylate Binding Protein 5 in these cell clusters, demonstrating an anti-viral response.^34^ Additionally, IGF1R was upregulated in adult Ciliary Cells (Clusters 1 and 2) and ionocytes and tuft cells, whereas in pediatric HNOs, IGF1R expression was detected across nearly all clusters, with peak expression in ciliated cells. Furthermore, RSV infection also led to a robust increase in interferon receptor gene expression in both adult and pediatric HNOs (Supplementary Figure 3A and B). Interestingly, while we did not observe major changes in *CX3CR1* expression, we detected an up-regulated expression of CX3CL1, particularly in RSV-infected cells. Fractalkine (CX3CL1) is not a known receptor for RSV but rather a potential competitor for binding to the CX3CR1, triggering an inflammatory response.^35^ Other known RSV-associated host factors, including *EGFR*, *ABCE1*, and *HSPG2*, displayed variable expression across the clusters but no striking differences were observed in either adult or pediatric HNOs (Fig. 2C and G).

Taken together, RSV appears to take advantage of several different host proteins differentially expressed on the epithelial cell populations that vary between adult and pediatric HNOs for efficient virus entry and infection.

### RSV preferentially infects primary ciliary cells and mucociliary cells in both adult and pediatric HNOs

**Fig. 3.**
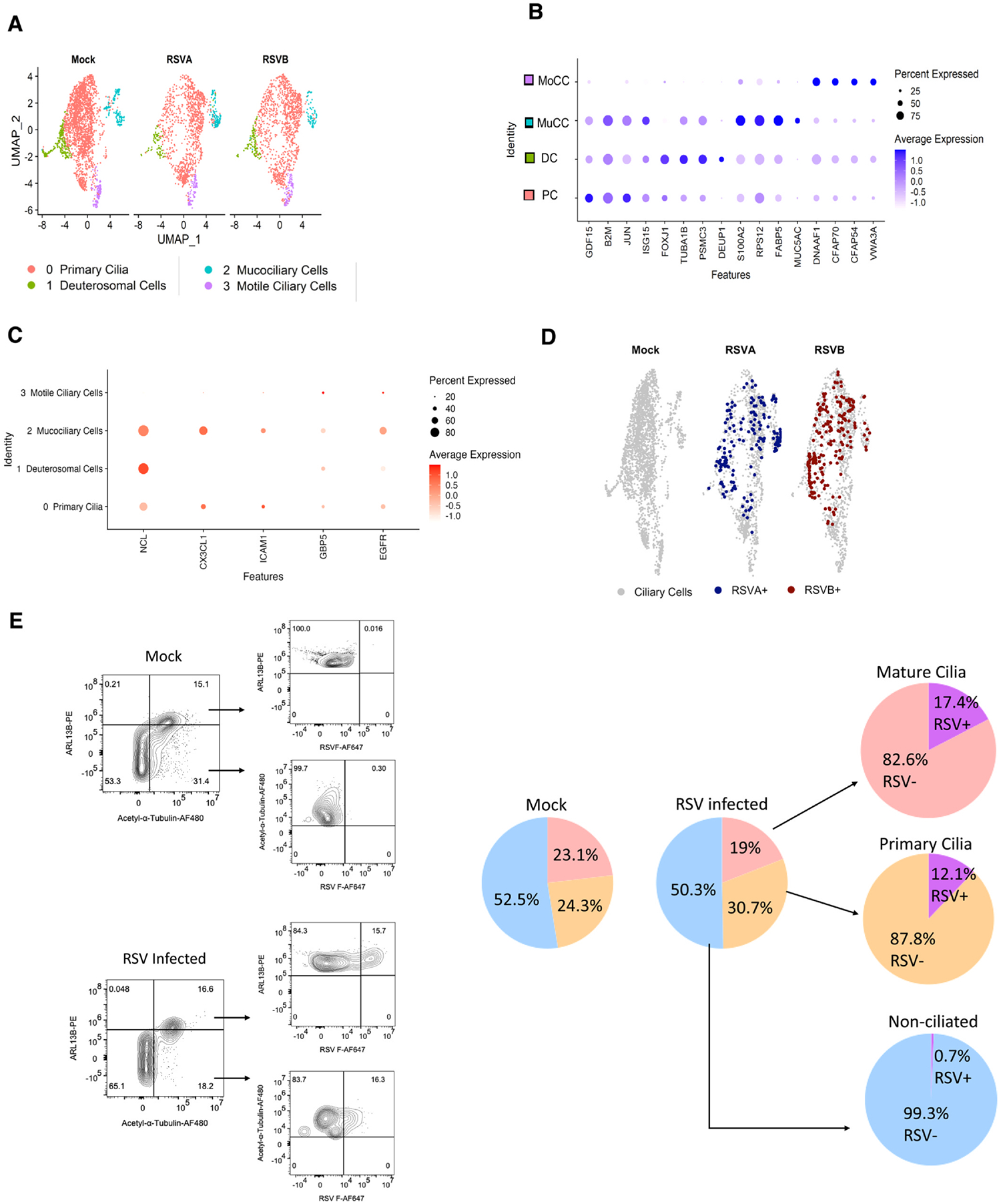

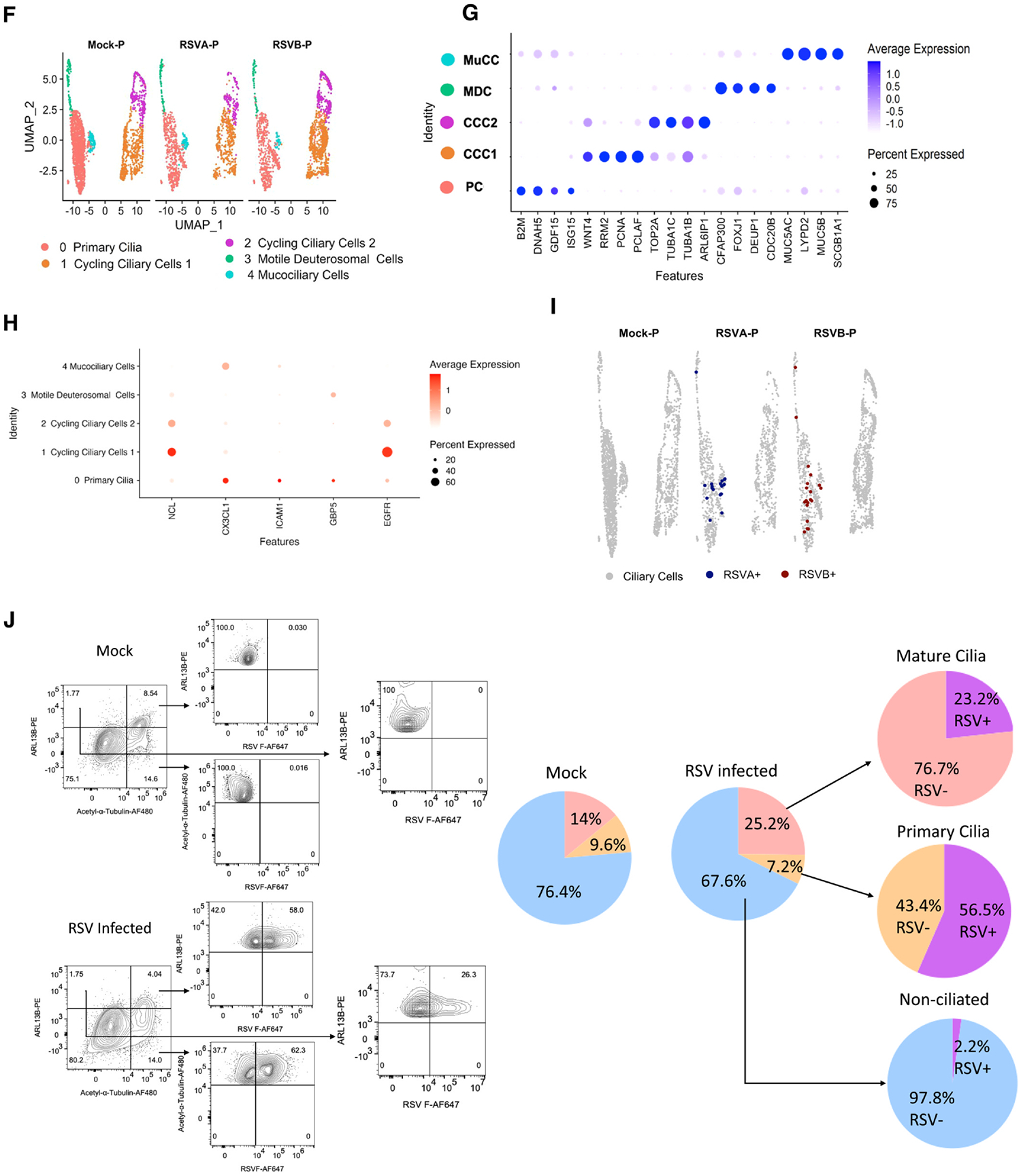
Sub-clustering Analysis Reveals Unique Ciliary Cell Subpopulations and RSV Tropism in Adult and Pediatric HNOs: (A) UMAP of subclustered ciliary cells from uninfected and RSV-infected adult HNOs. (B) Dot plots illustrate marker gene expression patterns used to define sub-ciliary cell clusters. Marker genes include MoCC (motile ciliary cells), MuCC (mucociliary cells), DC (deuterosomal cells), and PC (primary cilia). Dot size represents the percentage of cells expressing each marker, while color intensity (ranging from light blue to navy) reflects mean expression levels. (C) Selected up-regulated hypothesized RSV receptor genes in ciliary cell sub-clusters. (D) Distribution UMAP of ciliary cells containing RSV genes (NS1, NS2, N, and P). (E) Differential susceptibility of ciliary subpopulations to RSV infection in adult HNOs. Left: Representative spectral flow cytometry plots showing gating strategy to identify ciliated cell subtypes. Subsequent gating of RSV F protein expression was used to determine infection within each population. Right: Pie charts summarizing the distribution of ciliary and non-ciliary cell populations in mock- and RSV-infected adult HNOs. (F) UMAP of subclustered ciliary cells from uninfected and RSV-infected pediatric HNOs. (G) Dot plots illustrate marker gene expression patterns used to define sub-ciliary cell clusters. Marker genes include MuCC (mucociliary cells), MDC (Motile deutrosomal cells) DC (deuterosomal cells) CCC1(Cycling ciliary cell 1), CCC2 (Cycling ciliary cell 2) and PC (primary cilia). Dot size represents the percentage of cells expressing each marker, while color intensity (ranging from light blue to navy) reflects mean expression levels (H) Selected up-regulated hypothesized RSV receptor genes. (I) Distribution UMAP of ciliary cells containing RSV genes (NS1, NS2, N, and P). (J) Differential susceptibility of ciliary subpopulations to RSV infection in pediatric HNOs. Left: Representative spectral flow cytometry plots showing gating strategy to identify ciliated cell subtypes. Subsequent gating of RSV F protein expression was used to determine infection within each population. Right: Pie charts summarizing the distribution of ciliary and non-ciliary cell populations in mock- and RSV-infected pediatric HNOs.

Ciliogenesis plays a critical role in maintaining the homeostasis of cilia formation and the development of mature motile ciliary cells.^36^ Mature and motile ciliary cells in the airway epithelium are important orchestrators of the mucociliary clearance process and ensure the removal of mucus and debris. To better understand the cellular heterogeneity of ciliary cells in HNOs, we performed high-resolution sub-clustering of the ciliated cell population (Fig. 3). Our analysis revealed four distinct subpopulations of ciliary cells in adult HNO (primary, deuterosomal mucociliary and motile) and five sub-populations (primary, cycling ciliary 1 and cycling ciliary 2, motile deuterosomal and mucociliary) in pediatric HNOs. To date, the ciliary cells of the airway epithelium is broadly classified as deuterosomes and multiciliated cells using the markers FOXJ1, DEUP1, CDHR3, and DNAH5.^26,27^ In our study, both adult and pediatric HNOs expressed previously defined deuterosomal markers FOXJ1, DEUP1 (Cluster 1, adult HNO; cluster 3, pediatric HNO) (Fig. 3A and B, F and G). In addition, we identified additional sub-populations of ciliary cells, defined by unique expression of specific markers. In both adult and pediatric primary cilia population using unique expression of markers such as: GDF15, B2M, ISG15 (cluster 0) that have a role in sensing the environment, chemical, biological or mechanical stimuli^37^ (Fig. 3A and B, F and G). Another sub-population of ciliary cells- mucociliary cells defined by the expression of both ciliary cell markers and mucus secretion were present in both adult and pediatric HNO: RPS12, FABP5 and Muc5AC in adult (Fig. 3A and B) and Muc5AC, LYPD2, Muc5B, and SCGB1A1 in pediatric HNO (Fig. 3F and G).

Interestingly, in pediatric HNOs, we noticed a distinct group of cell clusters with a transcriptional signature of ciliogenesis (WNT4, PCNA, PCLAF, TOP2A, and TUBA1C), and these clusters are named cycling ciliary cells 1 and 2 (Fig. 3A and B, F and G). Adult HNO had a cluster representing motile and functional beating cilium defined by markers DNAAF1, CFAP70 and CFAP54, and these are termed motile ciliary cells. However, the motile ciliary cell cluster of pediatric HNOs was distinct from adults due to the presence of deuterosomal cell marker, DEUP1, and hence labeled as motile deuterosomal cells. (Fig. 3A and B, F and G).

Prior studies suggest that RSV primarily targets ciliary cells in the airway epithelium^8,20^ and furthermore in our previous studies we have demonstrated evidence for ciliary damage induced by RSV in HNOs.^25^ Hence, to determine how these subclustered ciliary cells respond to RSV infection, we analyzed the expression of RSV genes and potential RSV host receptors in this subpopulation at a higher resolution. Fascinatingly, we observed expression of four major RSV genes (NS1, NS2, N, and P) of both genotypes in primary ciliary cell clusters, indicating active viral infection and viral replication localized predominantly to the primary ciliary cell cluster in both adult and pediatric HNO (Fig. 3D and I, and Supplemental Figures 5 and 6). Replication of RSV in primary ciliary cells has not previously been reported and is a novel finding in RSV cell tropism. We further validated that primary cilia are the target of RSV replication using multi-spectral flow cytometry at 5 dpi. We identified different ciliary cell populations in both adult and pediatric HNOs as ciliated – Primary cilia (Arl13b+ and acetylated α-tubulin+) and mature cilia (Acetylated α-tubulin+) and non-ciliated cells (Arl13b- and Acetylated α-tubulin-).^36,38^ We found heterogeneity in ciliated cell populations in both mock and RSV-infected HNOs. In concurrence with our transcriptomic results, ciliated cells were infected by RSV, with primary cilia targeted by RSV predominantly in pediatric as compared to adult HNOs (Fig. 3E and J). Overall, our single-cell transcriptomic and multi-spectral flow cytometry data clearly indicate differential susceptibility and age-related tropism of the ciliary population to RSV infection.

Consistent with RSV infection of primary cilia, we also noticed that the expression of CX3CL1 and ICAM1 receptors was enriched in the primary ciliated cell cluster compared to other ciliary cell sub-clusters, although ICAM1 but not CX3CL1 is a potential receptor for RSV (Fig. 3C and H). The expression of different known RSV receptors varied across various ciliary cell clusters. For example, NCL was highly enriched in the deuterosomal cluster in adults, followed by the mucociliary and primary cilia clusters (Fig. 3C). In pediatric HNOs, NCL was enriched in the cycling ciliary clusters (Fig. 3H). EGFR expression was enriched in the cycling ciliary cluster of pediatric HNOs and the mucociliary cluster of adult HNOs (Fig. 3C and H).

Since a higher expression of RSV gene transcripts was found localized to the primary cilia cluster (Supplemental Figures 5 and 6), using GSEA (gene set enrichment analysis), we examined the changes in hallmark pathways [(Molecular Signatures Database (MSigDB)], and there were differences in the pathways across the ciliary subsets. Particularly, we observed higher enrichment of antiviral, inflammatory and apoptotic pathways in the primary cilia subcluster over others. It was notable that the downregulation of cell cycle and ciliogenesis pathways were more prominent in the cycling ciliary cells of pediatric HNOs (Supplementary Figure 4), however these cells were also infected with RSV based by their increased virus gene copy numbers (Supplementary Figures 5 and 6). Expression of Guanylate Binding Protein 5 with known antiviral activity against RSV was widespread in adult ciliary cells but was more specific to primary cilia and motile deuterosomal cells in pediatric HNOs (Fig. 3C and H). Similarly, CX3CL1 or fractalkine, a known inflammatory mediator, was enriched in the primary cilia cluster compared to other ciliary cell subclusters, suggesting that the primary cilia cells are generating antiviral and inflammatory signals upon RSV infection (Fig. 3C and H). These findings are also supported by pathway analysis with primary cilia subpopulations enriched for antiviral, inflammatory and apoptotic signaling (Supplementary Figure 4).

### RSV infection increases goblet cells, decreases club cells, and induces airway epithelial composition rearrangement in HNOs

**Fig. 4.**
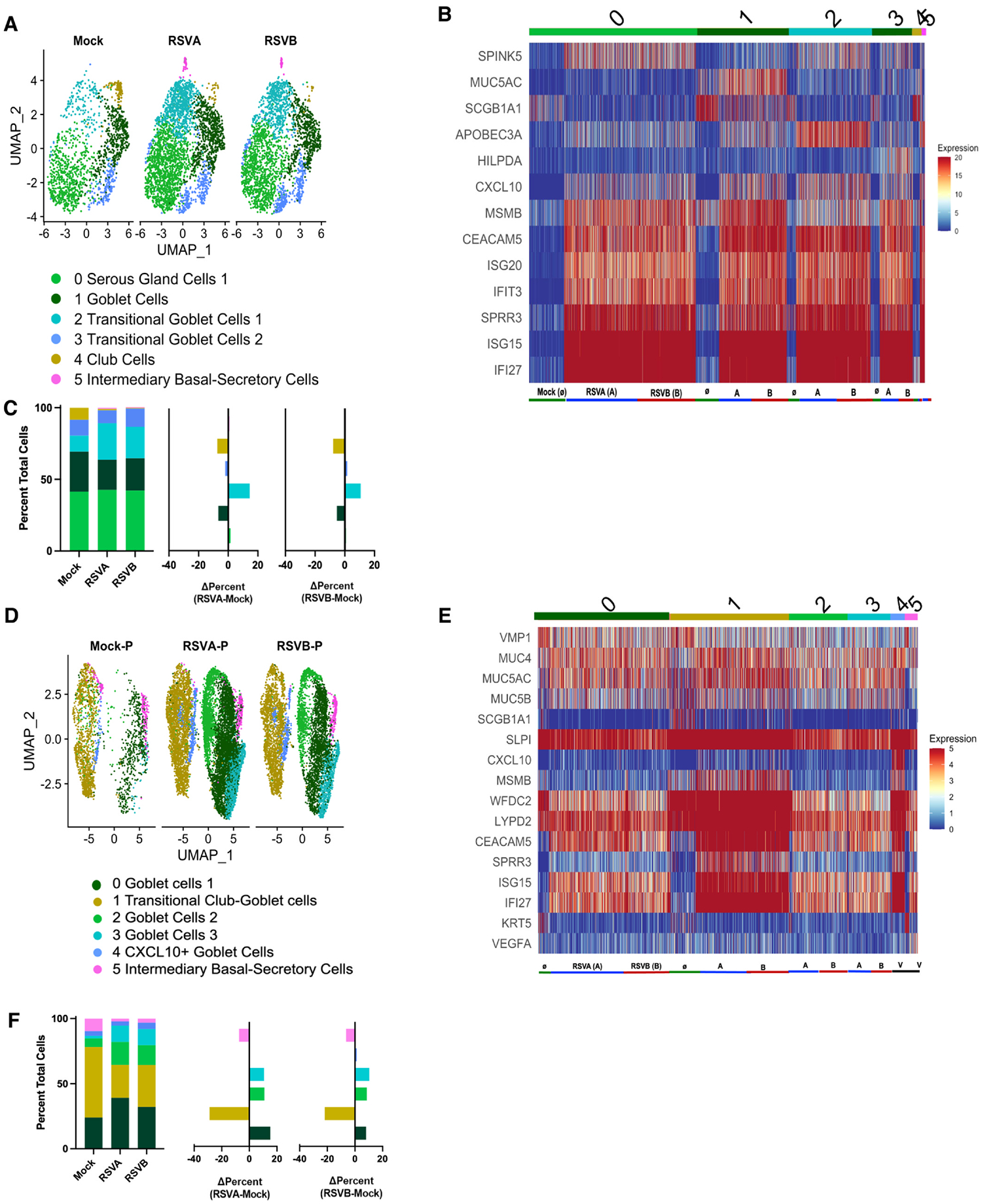
RSV-Induced Changes in Secretory Cell Populations Drive Airway Epithelial Remodeling in HNOs: (A) UMAP of subclustered secretory cells from uninfected and RSV-infected adult HNOs. (B) Heatmap of selected genes within the secretory cell subcluster of adult HNOs. (C) Quantification of percent cells per cluster (Left) and difference in percent (infected–uninfected) in secretory cell clusters (Right) (D) UMAP of subclustered secretory cells from uninfected and RSV-infected pediatric HNOs. (E) Heatmap of selected genes within the secretory cell subcluster of pediatric HNOs. (F) Quantification of percent cells per cluster (Left) and difference in percent (infected–uninfected) in secretory cell clusters (Right).

Increased mucus secretion is a hallmark feature for RSV infection and severity from RSV infection is often associated with increased mucus secretion.^15,19–21,25,37^ Particularly in pediatric subjects, it can cause severe airway obstruction, acute respiratory distress, and often leads to hospitalization.^2,5,15,23^ Despite these observations and associations, the detailed molecular mechanism of how RSV infection induces increased mucus secretion is unclear. Specifically, is it just an up-regulation of mucus production in the goblet cells, or does RSV alter airway epithelial cellular composition and increase the number of goblet cells? To investigate this, the secretory cell population was further clustered into six unique subclusters in adult and pediatric HNOs based on their transcriptional profile (Fig. 4A and D). Overall, we noticed a significant increase in the total number of secretory cells in RSV-infected HNOs (Supplemental Sheet 11). Goblet cell marker, MUC5AC expression was restricted to the goblet cell sub-cluster (cluster 1) in adult HNO, but several sub-clusters (clusters 0, 1, 2, 3, & 4) of pediatric HNO expressed MUC5AC (Fig. 4B and E). SCGB1A1, club cell marker was predominantly found in mock HNOs, but upon RSV infection by either genotype, a shift from SCGB1A1+ club cell to a MUC5AC+ secretory cell is noticeable (Fig. 4B and E). This is true for both adult and pediatric HNO, suggesting a potential change in epithelial cell composition induced by RSV infection. The adult HNOs revealed a unique secretory cell sub-cluster, serous gland cells (Cluster 0) marked by expression of serine protease inhibitor Kazal-type 5 (SPINK5), also known as lympho-epithelial Kazal-type related inhibitor (LEKTI) (Fig. 4A and B). SPINK5 is cleaved into functional fragments by the protease furin, the same protease known to activate the F protein of RSV into a fully activated form, the preF conformation.^39^ Serine protease inhibitors are known to have antiviral activity against RSV, especially early in infection.^40^ Remarkably, the SPINK5 expression is upregulated in the presence of RSV, suggesting an antiviral role of these cells.

Next, we investigated the changes induced by RSV infection in the goblet cell population and other secretory cell types of airway epithelium. There is an overall increase in the number of goblet cells in response to RSV infection by either genotype in both adult and pediatric HNOs [cluster 1, adult HNO; cluster 0, pediatric HNO] (Fig. 4C and F). In contrast, the club cell population of adult HNOs decreased in response to RSV infection by either genotype. The data suggest a transition to a dominant MUC5AC+ goblet cell population consistent with epithelial reconstruction from RSV induced transcriptional pathways (Fig. 4C). This is in concurrence with our prior study which showed an increase in goblet cells with RSV infection in both adult and pediatric HNOs.^24^ Changes in expression of putative goblet cell markers, MUC5AC and other goblet cell markers, upon RSV infection in pediatric HNOs suggested that overall goblet cell phenotypes increase in pediatric HNO (Fig. 4D and F). The specific increase in MUC5AC + goblet cells combined with the total decrease in club cells in adult HNOs but a relative decrease in pediatric HNOs (Fig. 4C and F) implies that the RSV infection of the airway epithelium stimulates the transition of the other secretory cells into a dominant goblet cell population. Interestingly, in addition to these changes, there was a unique group of goblet cells that showed high levels of CXCL10 expression in the pediatric HNOs, further indicative of pro-inflammatory goblet cells specific to the pediatric population. High levels of CXCL10 early during infection have been associated with protection against severe RSV infection (Fig. 4D and E).^41,42^

### RSV infects basal cells and ionocytes of pediatric HNOs

**Fig. 5.**
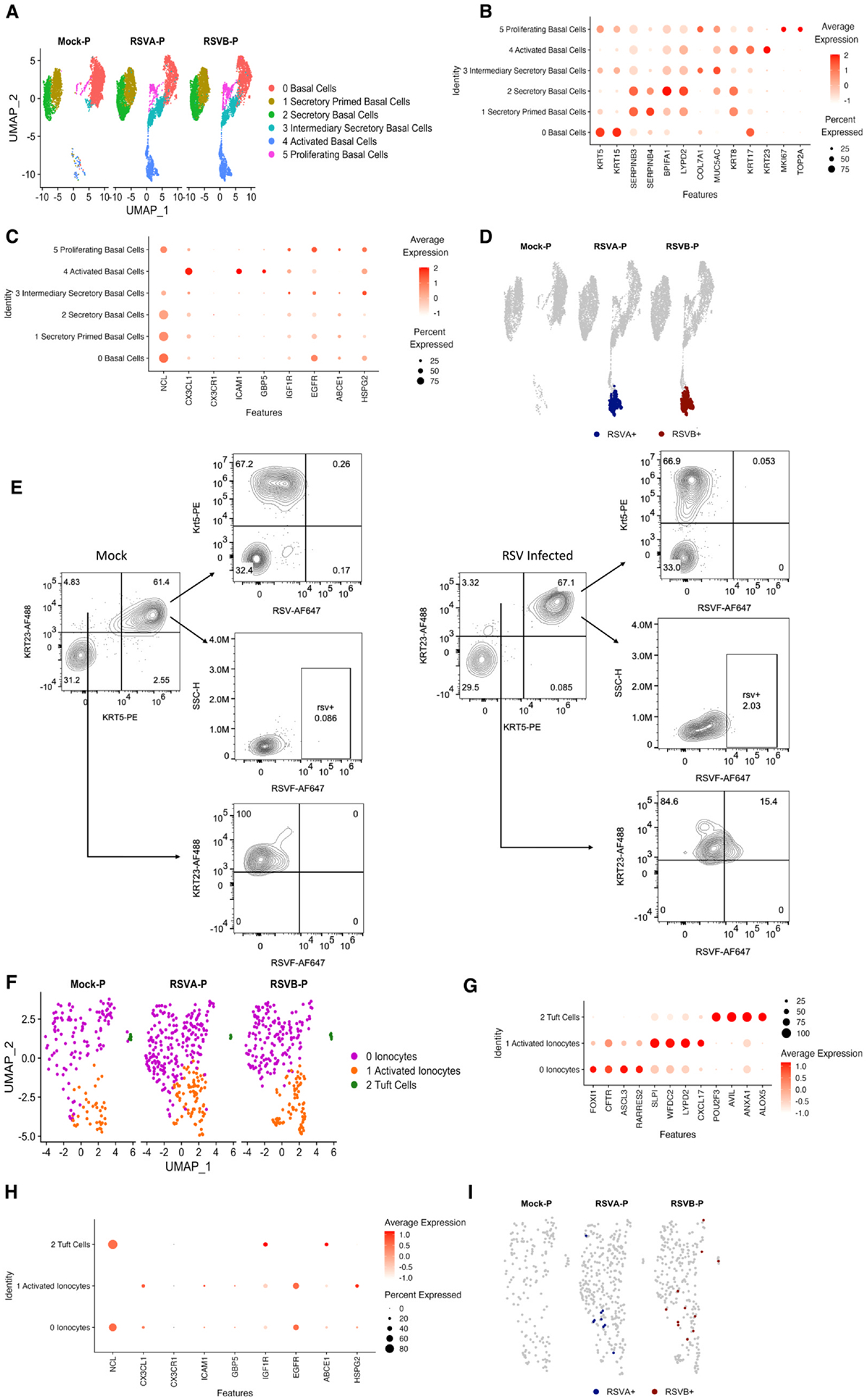
RSV Targets Activated Basal Cells and Ionocytes in Pediatric HNOs: (A) UMAP of subclustered basal cells from uninfected and RSV-infected pediatric HNOs. (B) Dot plots show marker genes used to define sub-basal cell clusters, with dot size representing the percentage of cells expressing each marker and color intensity (salmon to red) indicating mean expression levels. (C) Selected up-regulated hypothesized RSV receptor genes in basal cell sub-clusters. (D) Distribution UMAP of basal cells containing RSV genes (NS1, NS2, N, and P). (E) Flow cytometry contour plots showing the expression of basal cell markers KRT5 and KRT23, and RSV F protein in mock and RSV-infected pediatric HNOs. (F) UMAP of subclustered ionocytes and tuft cells from uninfected and RSV-infected pediatric HNOs. (G) Dot plots show marker genes used to define sub-ionocytes and tuft cell clusters, with dot size representing the percentage of cells expressing each marker and color intensity (salmon to red) indicating mean expression levels. (H) Selected up-regulated hypothesized RSV receptor genes in ionocytes and tuft cell sub-clusters. (I) Distribution UMAP of basal cells containing RSV genes (NS1, NS2, N, and P).

*Persson et al*. demonstrated that basal cells of the airway epithelium can be infected by RSV in a mechanical injury model.^15^ However, since the infection was facilitated by an artificial wound-induced system, questions remain about the natural susceptibility of basal cells to RSV under physiological conditions. Despite this, understanding pthe ermissiveness of other airway cell types to RSV, specifically the progenitor cell type, the airway basal cells, remains a critical area of investigation in elucidating RSV pathogenesis. Infection of additional cell types in the airway epithelium could explain the severity associated with RSV infection in certain individuals who have increased cell plasticity or chronic airway phenotypes. In our dataset, in addition to finding RSV genes in the ciliary cells, we also found RSV genes of both genotypes in basal cell clusters of pediatric HNOs [cluster 9] but not conclusively in adult HNOs (Fig. 2C and G). By sub-clustering the basal cell population of pediatric HNOs, we found high viral gene copies of RSV (NS1, NS2, N, and P) restricted to a specific sub-cluster of basal cells, Activated Basal Cells [AcBC] (Fig. 5A and D). These AcBC have enriched expression of KRT23 (a proliferative marker for expansion of cells) in addition to KRT8 and KRT17 expression, which indicates the transition of basal cells to an aberrant pro-fibrotic mesenchymal transition (Fig. 5B).^43^ Consistent with the earlier association of CX3CL1 and ICAM1 expression in RSV-infected cells, these AcBC also demonstrated enriched expression of CX3CL1 and ICAM1 in comparison to the other basal cell sub-population (Fig. 5C).

For further confirmation of AcBC infection, the pediatric HNOs infected with RSV were stained for Krt5 and Krt23 antibodies (Fig. 5E). Our analysis by flow cytometry revealed a population of cells expressing both KRT5 and KRT23; however, RSV positivity (~2.03%) was observed exclusively within the KRT23+ subset, indicating a low but detectable level of infection. Notably, no RSV+ cells were detected within the KRT5+ population, suggesting that KRT5+ expressing basal cells may be less permissive to RSV. Strikingly, a larger RSV+ subpopulation (~15.4%) was identified within KRT23+ cells, further supporting the notion that a specific subset of basal cells marked by KRT23 is susceptible to RSV infection (Fig. 5E). Overall, our data supports the hypothesis that airway basal cells are susceptible to RSV infection, though the infection rate is relatively low in the HNO model. This aligns with prior findings using injury-induced models, but here shows direct evidence without mechanical wounding—suggesting a degree of intrinsic permissiveness to RSV in a basal cell subset with expression of KRT23.

GSEA analysis of these basal cell subclusters exhibited an anti-viral response characterized by induction of ISG15, antiviral interferon pathways and inflammatory pathways specifically in the AcBC (Supplementary Figure 7A–D). In stark contrast, the basal cell sub-clusters of adult HNOs showed no RSV genes of either genotype, and consistently, the expression of ICAM1 was not enriched (Supplementary Figure 7A and C). Yet we noticed an antiviral response in the basal cell clusters of adult HNO, indicating a global antiviral response from uninfected basal cells, potentially due to bystander effects.

Ionocytes are rare epithelial cell types of airway epithelium and are marked by the presence of FOXI1.^27^ Like our findings of RSV genes in the basal cells of pediatric HNO, we detected RSV genes in the ionocyte/tuft cell clusters of pediatric HNOs [cluster 11] but not adult HNOs (Fig. 2C and G). Hence, we subclustered the ionocytes/tuft cell cluster of pediatric HNO at a higher resolution, and our results show three individual clusters: ionocytes (FOXI1, CFTR, ASCL3), activated ionocytes (low expression of FOXI1, CFTR and high expression of LYPD2, EFDC2, SLPI) and tuft cells (POU2F3, AVIL, ALOX5) (Fig. 5F and G). Within these sub-clusters, RSV genes were enriched specifically in the ionocytes but not tuft cells, and consistently higher expression of EGFR, ICAM1, and HSPG2 were also noticed in the activated ionocytes cluster (Fig. 5H). An increase was also seen in the subpopulations of ionocytes in response to RSV infection (Fig. 5I). We observed a strong antiviral response via induction of ISG15, IFI27, IFI6 genes and interferon pathways in the ionocyte clusters indicative of viral infection (Supplementary Figure 8A–D). All this evidence suggests that AcBCs and ionocytes could be novel and uncommon cell types of airway epithelium that are infected by RSV in children and may play an underappreciated role in its pathogenesis.

## Discussion

In the last few years, a few studies have reported single-cell genomic and transcriptomic analysis on human airways in both healthy and disease-compromised individuals (both infectious and non-infectious).^26,27,44,45^ Our study provides a first comprehensive reference for a single-cell atlas for human nose organoids obtained from an adult and a pediatric subject. Altogether, we profiled 84,255 cells, capturing both the baseline gene expression landscape as well as RSV-induced transcriptional changes in HNOs, confirming some of our novel findings during RSV infection using orthogonal approaches. This dataset provides a unique opportunity to compare and contrast the gene expression changes (both uninfected and RSV-infected) at a single cell level between an adult and a pediatric subject. Previous studies using human lung, nasal brushing, and tracheal samples have identified major epithelial cell types, including ciliated cells, basal cells, goblet cells, club cells, pulmonary neuroendocrine cells, and ionocytes.^27,46^ Consistently, our HNOs exhibited hallmark features of bronchial epithelium and included all major airway epithelial cell types, with the exception of pulmonary neuroendocrine cells (Fig. 1). These findings demonstrate that HNOs recapitulate the key cellular composition of the human bronchial airway, supporting their utility as a physiologically relevant model to study both upper and lower airway epithelial responses. Furthermore, use of our non-invasive HNO model, provides an easily approachable, yet a powerful platform to rapidly increase scalability and provide high-throughput data to generate novel information in the context of understanding age differences in airway epithelial responses.^24^ This study further characterizes fundamental differences in the airway epithelium of HNOs derived from adult and pediatric individuals, delineating distinct variations in epithelial composition and gene expression profiles (Fig. 1).

We first observed marked differences in the cellular proliferation and differentiation capacities of the airway epithelium between adult- and pediatric-derived HNOs (Fig. 1A and B). The expression of Ki67, a marker of cellular proliferation, was reduced in both adult and pediatric HNOs following infection with RSV, compared to mock-treated controls; however, only in adult HNOs was a marked and significant reduction observed during infection with both contemporaneous RSV genotypes. This decline suggests that RSV infection or the host response to RSV infection suppresses epithelial cell proliferation in airway tissues. Notably, the reduction in Ki67-positive cells was more pronounced in adult samples, in response to both RSV/A and RSV/B, indicating a potentially age-dependent effect of RSV on host cellular turnover or repair responses (Fig. 1E). Alternatively, the host cell in response to RSV infection might shut down its cell cycle and favor an apoptotic pathway as suggested by our pathway analysis of primary cilia cell subpopulation for control of virally infected epithelial cells.^24,47^ Pediatric HNOs also showed a decrease in proliferation, though to a lesser extent, which may reflect developmental differences in epithelial regenerative capacity or distinct immune modulation in younger individuals. Important variations in gene expression were identified when comparing the populations of types and number of basal cells and secretory cells between adult and pediatric HNOs. Despite these differences, the majority of epithelial cell types and transcriptionally defined clusters were shared across both adult and pediatric HNOs (Fig. 1C and D). These common cell clusters expressed similar canonical marker genes; however, the expression levels and distribution of these markers were broader and more prominent in pediatric HNOs (Fig. 1B and D). Importantly, these molecular distinctions were also reflected in the epithelial phenotype, with pediatric HNOs displaying a higher proportion of proliferative cells and a more dynamic basal cell population (Fig. 1B and E).

Secondly, to expand our fundamental understanding of RSV infection, cell tropism, and pathogenesis, we infected these adults and pediatric HNOs with both contemporaneous genotypes of RSV at an MOI of 0.01 (RSV/A/ON and RSV/B/BA) and examined epithelial response to RSV at 5 days post-infection (dpi). These experiments revealed consistency in RSV infection between both genotypes with high levels of viral RNA in specific sub-populations of HNOs including ciliary cells (both adult and pediatric), basal cells and ionocytes (Fig. 2C and G), indicating RSV-infected cells. Using multi-spectral flow cytometry in HNO lines distinct from those used in the scRNA-seq analysis, we further confirmed active RSV infection in ciliary cells, including the newly identified primary cilia cells of the nasal epithelium, as well as in Krt23-positive basal cells. Flow cytometry confirmed productive infection as evidenced by RSV F antigen detection in multiple epithelial cell subtypes, consistent with the transcriptional evidence from scRNA-seq. These findings were reproducible across independent donor-derived pediatric lines, underscoring that RSV infection of multiple epithelial cell types is a robust and donor-independent phenomenon. The GSEA analysis identified multiple antiviral and pro-inflammatory pathways enriched and up-regulated at higher levels relative to mock-infected cells, specifically in the cell clusters that had high levels of viral gene copies (Supplemental Figure 1 and Supplemental Sheet S3-S6).

In our previous study^24^ we showed robust secretion of type III interferons and pro-inflammatory cytokines with RSV infection, consistent with the interferon-stimulated gene expression and inflammatory pathway activation observed here by scRNA-seq. The cytokine secretion patterns reported in our prior study align well with the enrichment analysis identified in the current analysis, together supporting a strong antiviral and inflammatory response across both adult and pediatric HNOs.

Numerous genes associated with ciliogenesis, metabolism, oxidative phosphorylation, and cell cycle regulation were down-regulated by infection (Supplemental Sheet S7-S10). Multiple genes involved in drug metabolism/xenobiotic metabolism, hypoxia regulation, and epithelial-mesenchymal transition were also down-regulated by RSV infection. These alterations in specific cell types induced by RSV raises an intriguing question on how these cell clusters may respond to hypoxic environment or therapeutics in comparison to uninfected cells.

Repeatedly, it has been suggested and indicated that the apical ciliary (multi-ciliated) cells of airway epithelium are the primary site for RSV replication.^8,10,19,20,25,31–33^ Yet no study has looked within the ciliary cell subpopulations, which ciliary cell types are more permissive to RSV infection, and which cell receptors dictate this tropism. In our dataset, we identified 4 distinct clusters of ciliary cells including primary ciliary cells, deuterosomal cells, mucociliary cells and motile/matured ciliary cells (Fig. 3). It was interesting to note the HNOs also revealed the presence of recently discovered hybrid mucociliary cells that respond to an assault.^14,48^ During an assault or injury, these cells seem to preferentially lose ciliogenic function and can potentially hinder the regeneration of ciliated cells.^45^ Based on gene copy numbers, RSV seems to preferentially infect the primary ciliary and mucociliary cells and to a lesser degree the motile/matured ciliary cells (Fig. 3). This is a previously unidentified cell tropism to RSV infection by specific sub-populations of ciliary cells.^33^ Using multispectral flow cytometry, we characterized ciliated cell populations in both pediatric and adult human nasal organoids (HNOs), distinguishing between primary cilia, mature cilia, and non-ciliated cells. We observed notable heterogeneity among ciliated populations under both mock and RSV-infected conditions. Importantly, RSV preferentially infected ciliated cells, with a striking bias toward primary cilia in pediatric HNOs, consistent with previous reports highlighting enhanced vulnerability of pediatric airways to RSV. In adult HNOs, the primary cilia and multi-ciliated cells appeared equally receptive to RSV infection. This age-dependent tropism likely reflects developmental differences in epithelial cell maturation and innate immune competence, positioning primary ciliated epithelial cells as a major viral target in early life. Interestingly, there is evidence of primary monocilia-targeted effects from Zika virus studies.^49^ Our findings underscore the complex and cell type–specific tropism of RSV within the airway epithelium, revealing both developmental and cellular determinants of susceptibility.

Our results also suggest that ICAM1, a well-known receptor for human rhinovirus, may also serve as a potential receptor for RSV, and this could influence the cell tropism for RSV in primary ciliary cells and mucociliary cells.^50^ The primary cellular receptor for attachment by the G protein of RSV that is located on the apical surface is CX3CR1. Interestingly, HSPG which is also upregulated during RSV infection is the primary receptor for the G protein in continuous cell lines, but it is not found on the apical surface of the airway epithelium.^51,52^ HSPG, however, is found on the basal cells and may play an important role as a G protein receptor in RSV infection of non-ciliated cells. On the other hand, the F protein of RSV appears to have various known cellular receptors including nucleolin, IGFR1, EGF, and ICAM1 for binding followed by viral entry.^31^ Such a strategy might allow the virus to efficiently infect the respiratory epithelium during various stages of remodeling in response to different stresses or development.^24^ However, the specific sub-cellular tropism in nasal epithelial cells remains unknown. This study provides new insights into how the distribution and expression of receptors across various cell types may influence RSV tropism within the cell population.

In fact, these primary and mucociliary cells showed higher levels of antiviral response and pro-inflammatory response compared to other ciliary subsets (Supplemental Figure 4). Interestingly, in both adult and pediatric HNOs, we noticed that TNFα signaling pathway was upregulated only in ciliary cell cluster having high levels of RSV gene copies. Consistent with this finding, in our previous HNO work, we observed higher levels of TNFα in RSV-infected cells relative to mock,^25^ similar to what was observed in our clinical studies,^41,53^ and our current data provides evidence that it could be the ciliary cells of airway epithelium that produce and secrete TNFα. An intriguing aspect of our study is the potential involvement of TNFα signaling via NF-κB in mediating the distinct inflammatory responses observed between adult and pediatric HNOs. Understanding these age-specific dynamics is crucial as they not only elucidate the mechanisms underlying disease severity but also highlight the need for age-targeted therapeutic interventions.^54,55^ This differential signaling also underscores the importance of designing RSV therapies and vaccines that address the distinct molecular and cellular environments of adult and pediatric airway epithelia. However, future studies are needed to validate these findings to explore the possibility of targeted immunotherapeutic.

A study by *Persson et al*.^15^ demonstrated that basal cells of the airway epithelium could also be infected by RSV through a mechanical injury model. Consequently, an unanswered and lingering question is whether RSV can infect basal cells, and if so, how do these cells respond to infection and changes/influence disease progression? Moreover, multiple studies have shown evidence that assault to basal cells, either by chemical injury or through infections, can cause dysregulation of basal cell activity, cellular proliferation, and differentiation. This disruption can cause aberrant expansion, self-renewal process resulting in fibrosis of airway epithelium, chronic inflammatory diseases such as chronic obstructive pulmonary disease (COPD) and even result in neoplasia.^16,56,57^ In our previous study^24^ we observed that pediatric HNOs nearly had twice as much basal cells as adults and hence identifying if RSV can truly infect basal cells would be critical to evaluate its role in causing exacerbation of lung diseases.^25^

Using multispectral flow cytometry, we examined whether RSV infects AcBCs by analyzing the expression of KRT5 and KRT23 in pediatric HNOs. While a dual-positive KRT5+/KRT23+ basal population was present, RSV infection was restricted to the KRT23+ subset. This suggests that classical basal cells marked by KRT5 may be less permissive to RSV, while KRT23 expression identifies a distinct basal subset that is more susceptible to infection. These findings are particularly noteworthy given that earlier studies demonstrated basal cell infection primarily in the context of epithelial injury. In contrast, our results provide direct evidence of RSV infection in basal cells within an intact, uninjured epithelium (Fig. 5D and E), supporting the idea that certain basal cell subsets possess intrinsic features that enable RSV entry and replication. Hence, our study for the first time provides evidence at the single-cell level that RSV can naturally infect airway basal cells. This specific affinity in certain populations of basal cells could contribute to the severity associated with RSV in specific individuals/age groups, thus emphasizing the role of the host in disease severity. A surprising finding in our study is that pediatric ionocytes were also susceptible to RSV infection (Fig. 5I). Altogether, these data provide evidence for novel cell tropism beyond the apical ciliary cells, and identifying these is critical for the development of effective treatment options and antiviral therapies. Especially, the development of alternative therapeutic strategies targeting host factors to combat viral entry.

A focus on secretory cell cluster via side-by-side comparison of the adult and pediatric HNOs demonstrates inherent differences, wherein adult HNOs show a defined and well-differentiated cellular phenotype. The secretory cells of adult HNOs are mainly composed of serous gland (SPINK5), club cells (SCGB1A1), and goblet cells (MUC5AC), and each of these clusters displayed its core gene signature distinctly (Fig. 4A and B). However, the pediatric HNOs, though demonstrating multiple secretory cell subclusters, the majority expressed MUC5AC and MUC5B expression, suggesting a transcriptional setting for multi-mucin producing mucosal secretory cells. Our prior HNO study comparing adult versus pediatric HNO clearly confirms the increased mucous-producing capacity of pediatric HNOs compared to adult HNOs both before and during RSV infection.^24^ Our current study sheds light on pediatric-specific physiological and pathological differences in airway epithelium and they are particularly important to consider when choosing the origin of HNO to distinguish age-specific responses to a disease phenotype. Second, we provide a detailed characterization of how the secretory cells respond to RSV infection that could explain some of the clinical observations of heightened severity to RSV infection in pediatrics. In our earlier study and as well as other have demonstrated that RSV induce goblet cell hyperplasia phenotype and this is significantly enhanced in the pediatric subjects in comparison to adults.^21,22,25^ Our results demonstrate that RSV infection upregulates the expression of MUC5AC specifically only goblet cells of adult HNOs (Fig. 4B: cluster 1, adult HNOs); whereas in the pediatric HNOs, RSV induced MUC5AC across all secretory cell types suggesting a shift in secretory cells toward a MUC5AC-dominated inflammatory state (Fig. 4E). This phenotype, combined with damage to ciliary epithelium and decrease in club cells both induced by RSV could explain why specifically pediatrics infected with RSV, are even more vulnerable to severity of RSV infection. Currently, maternal vaccination and monoclonal antibody immunoprophylaxis are prescribed to mitigate RSV infection during the first year of life. Our data suggest the possibility of targeting cytokine/chemokine receptor, CXCL10. We found a specific cluster of secretory cells in pediatric HNOs (Cluster 4) with an increased expression of CXCL10, suggesting a potential target for pharmacological interventions of infants with severe RSV infection. In support of this, a previous study has shown that immunotherapies against CXCL10 can significantly reduce disease severity, hyper secretion of mucus, airway hyperresponsiveness and impaired viral clearance.^58^ However, early and robust induction of CXCL10 may protect infants against severe RSV disease,^41,42^ suggesting timing of intervention may be critical for mitigating or enhancing disease severity.

Altogether, we believe our study has made a significant contribution by providing a comprehensive analysis of the transcriptomic profile of healthy adult and pediatric HNOs and airway epithelial response to RSV of both major contemporaneous genotypes at a greater resolution. A notable limitation of our study is the small sample size used for the single-cell sequencing analysis. Expanding the analysis using this information and further standardizations are required to draw more definitive and phenotypic conclusions on the age difference. Our analysis of the nasal epithelial response to RSV was limited to a single time point—5 dpi, based on our previous findings showing peak viral replication and cytokine response at this time.^24,25^ However, more extensive investigations incorporating additional time points and experimental conditions, such as RSV-targeted therapeutics, would enable deeper spatial and temporal profiling. Such studies could enhance our understanding of the differential vulnerability to RSV infection and epithelial remodeling across age groups. Nevertheless, our work has elucidated novel insights into RSV infection dynamics in both adults and pediatric HNOs and may hold the key towards the development of new therapies specific to various age groups and pave the way for personalized medicine.

## Materials and methods

### Human nose organoid (HNO) culture and RSV infection

HNO-ALIs were obtained from the Baylor College of Medicine 3D Organoid Core and cultured using standard conditions as described previously.^25^ The Institutional Review Board at Baylor College of Medicine approved the study protocol (H-46014 approval date 3/9/2021) to obtain the samples from which the organoid lines were established, and informed consent was obtained from each donor. Two HNO lines used for single-cell RNA seq were HNO 204 (Female, age 75) and HNO 9005 (Male, age 1.5 years). Additionally, two other HNO lines were used for validation experiments by flow cytometry they were HNO 918 (Female, age 45 years) and HNO 9003 (Female, age 0.8 years). Samples were processed by enzymatic digestion in AO medium with collagenase and amphotericin B, followed by filtration and washing to isolate epithelial cells. Cells were embedded in Matrigel and cultured in AO medium with penicillin-streptomycin-amphotericin (PSA), with medium replaced every 4 days. Mature HNOs were dissociated into single cells and seeded onto collagen-coated Transwells at 3 × 10^5^ cells/well to create air-liquid interface (ALI) cultures using PneumaCult-ALI medium. Differentiated ALI cultures were infected apically with RSV/A/USA/BCM813013/2013(ON) or RSV/B/USA/BCM80171/2010(BA) at an MOI of 0.01 for 1.5 h at 37 °C. Post-infection, cultures were maintained air-exposed, and samples were collected at 5 days post-infection (dpi) for single-cell RNA sequencing, flow cytometry or immunofluorescence to investigate RSV tropism and epithelial responses.

### Single-cell isolation and FACS for HNO and RSV-infected cells

At 5 days post-infection (dpi), Transwell cultures of mock- and RSV-infected HNOs were washed on the apical side with EDTA-PBS and carefully excised using a fine blade to avoid damage. Membranes were transferred into Eppendorf tubes containing 500 μL of Accutase and incubated at 37 °C for 40 min, with gentle pipetting every 10 min using a wide-bore pipette tip to facilitate dissociation using the previously published protocol.^59^ Dissociated cells were resuspended in AO medium and pipetted up and down 20–30 times to achieve single-cell suspensions while minimizing damage. The cell suspension was filtered sequentially through 70-μm and 40-μm Flowmi strainers into clean tubes to remove aggregates. Cell concentration and viability were assessed with Trypan Blue, targeting a concentration of 1000 cells/μL with > 90% viability and minimal clumps. For FACS, cells were stained with antibodies for epithelial (EpCAM+). Dead cells were excluded using DAPI staining, and single EpCAM+ cells were collected for downstream single-cell RNA sequencing.

### Single-cell RNA sequencing

Single-cell RNA sequencing was performed following an adapted protocol from Bomidi *et al*.^60^ for transcriptomic profiling of virus-infected organoid models. HNOs were enzymatically dissociated into single-cell suspensions with > 90% viability, confirmed by Trypan Blue staining. Single cells were sorted and processed for droplet-based library preparation using the 10x Genomics Chromium platform with the Single Cell 3′ Kit v2 or v3, depending on the batch. Libraries were prepared according to the manufacturer’s protocol, ensuring compatibility between v2 and v3 chemistries, and paired-end sequenced on the Illumina NovaSeq 6000 platform using an S2 flow cell to achieve approximately 50,000 reads per cell. Sequencing cycling conditions were as follows: Read 1: 28 cycles, Index 1: 8 cycles, Index 2: 0 cycles, and Read 2: 91 cycles. Data preprocessing, including unique molecular identifier (UMI) counting, clustering, and dimensionality reduction, was performed using Seurat for downstream analysis.

### Single-cell RNA sequencing and data processing

Sequencing reads from single-cell RNA sequencing experiments were aligned to the human genome (Homo_sapiens. GRCh38.106) and the RSV genome using the Cell Ranger pipeline (10x Genomics). Quality filtering and downstream analysis were performed using Seurat v4.2.0 in R v4.0. A total of 84,255 cells were retained after filtering, which excluded cells with greater than 20% mitochondrial gene content and regressed out mitochondrial gene expression during clustering to minimize confounding effects. Dimensionality reduction and clustering were performed using Uniform Manifold Approximation and Projection (UMAP), a preferred method for high-dimensional data visualization, as previously described.^27^ Sub-clustering was carried out for specific populations by reanalyzing the subset independently, regressing out mitochondrial content. For ciliary and basal cell subclusters, additional markers and pathway analyses were utilized following established approaches.^26,44,45,48,61,62^ Differentially expressed genes were identified using nonparametric Wilcoxon rank-sum tests, and pathway enrichment was inferred using Gene Set Enrichment Analysis (GSEA). For airway epithelial subclustering, methods from Jackson et al., Goldfarbmuren et al., Miller et al.,^44,48,61,62^ were integrated to improve identification and validation of cell states and transition markers.

### Immunohistochemistry

HNO-ALI cell lines were fixed in image-iT^™^ Fixative Solution (4% formaldehyde) [catalog number: FB002] for 15 min followed by dehydration in ethanol series (30%, 50%, and 70%, each 30 min at room temperature or overnight at 4°C). The transwell membranes were then embedded in paraffin and sectioned. For immunofluorescence staining, the sections were deparaffinized in Histo-Clear, followed by washes in an alcohol sequence (100 > 100 > 90 > 70%). Then the slides were rehydrated and exposed to heat-induced antigen retrieval in 10 mM citrate buffer pH (37). The sections were then washed in water for 5 min and blocked for 30 min in 2% bovine serum albumin (BSA) in blocking buffer (PBS). For dividing cells, the Ki67 antibody (1:500, Abcam, catalog number: ab15580) was used. Secondary antibody detection was by ImmPRESS^®^ Horse HRP anti-rabbit PLUS Polymer Kit (Vector Labs, catalog number: MP-7801). The percentage of dividing cells (Ki67+ stained cells) was compared to the total number of Hematoxylin+ cells.

### Intracellular staining and FACS analysis

For flow cytometry validation experiments, adult and pediatric HNOs lines (HNO918, HNO 9003) were infected with RSV/A as described above. HNOs were then washed with PBS and gently dissociated from culture wells using TrypLE Select (Thermo Fisher Scientific). The resulting cell suspensions were centrifuged at 500g for 5 min at 4 °C. Pelleted cells were transferred to 96-well U-bottom plates (Thermo Fisher) and stained for viability using the LIVE/DEAD^™^ Fixable Violet Dead Cell Stain Kit (Thermo Fisher) for 10 min at room temperature in the dark. Cells were then washed with FACS buffer (0.5% BSA, 1 mM EDTA in PBS) and permeabilized using the Cytofix/Cytoperm^™^ Fixation/Permeabilization Kit (BD Biosciences), following the manufacturer’s instructions. Immunostaining for intracellular proteins was performed using the following primary conjugated antibodies: KRT5 (1:5000, Abcam, catalog number: ab224985), KRT23 (1:100, Novus Biologicals, catalog number: NBP2–22590AF488), Arl13b (1:300, Santa Cruz, catalog number: sc-515784 PE), acetylated α-tubulin (1:100, Santa Cruz, catalog number: sc-23950 AF488), and RSV-F protein (1:100, Novus Biologicals, catalog number: NB100–63020AF647). Antibodies were incubated for 30 min on ice. After staining, cells were washed twice with BD Perm/Wash buffer and resuspended in FACS buffer prior to acquisition on a Cytek Aurora CS spectral flow cytometer. Data were analyzed using FlowJo software (BD Biosciences).

## Supplementary Material

Supplementary figure legends

Supplementary figures

## Appendix A. Supporting information

Supplementary data associated with this article can be found in the online version at doi:10.1016/j.jinf.2025.106617.
