# Supplementary material for: Single cell sequencing analysis of respiratory syncytial virus–infected pediatric and adult human nose organoids reveals age differences, proliferative diversity and identifies novel cellular tropism
Source: J Infect. Author manuscript; Available in PMC 2026 Mar 22. (PMC13006048; doi:10.1016/j.jinf.2025.106617)
Supplement: Supplementary figure legends [file NIHMS2150675-supplement-Supplementary_figure_legends.docx]

**Supplemental Figure 1: Hallmark Gene Set Enrichment Analysis of the adult and pediatric HNO cell subsets.**

Heatmaps show enrichment scores and adjusted p-values for hallmark gene sets across different cellular subsets. Grid colors represent the enrichment scores, with red indicating high enrichment and blue indicating low enrichment (NES: Normalized Enrichment Score, adjusted p < 0.05). (A) Heatmap of adult HNO cells infected with RSV/A/ON compared to mock. (B) Heatmap of adult HNO cells infected with RSV/B/BA compared to mock. (C) Heatmap of pediatric HNO cells infected with RSV/A/ON compared to mock. (D) Heatmap of pediatric HNO cells infected with RSV/B/BA compared to mock.

**Supplemental Figure 2: Dot plot of RSV infection related gene expression in adult and pediatric HNOs.**

Dot plots illustrate the expression of selected marker genes across epithelial cell subsets in (A) adult and (B) Pediatric HNOs. The size of each dot corresponds to the proportion of cells expressing the respective gene, while the color scale reflects the mean expression level (mock: white to gray; RSV/A: gray to navy blue; RSV/B: gray to red).

**Supplemental Figure 3: Dot plot of antiviral and interferon-related gene expression in adult and pediatric HNOs**

Dot plot showing expression of selected antiviral and interferon pathway associated genes across epithelial cell subsets in (A) adult and (B) pediatric HNOs. Dot size indicates the proportion of cells expressing each gene, while color intensity represents the mean expression level (mock: white to gray; RSV/A: gray to navy blue; RSV/B: gray to red).

**Supplemental Figure 4: Hallmark Gene Set Enrichment Analysis of the ciliary subset in adult and pediatric HNOs.**

The heatmap illustrates the enrichment scores and p-values for various hallmark gene sets across the different cellular subsets. The colors in the grids indicate the gene set's enrichment scores, where red signifies high enrichment and blue indicates low enrichment. (NES: Normalized Enrichment Score, adjusted p value < 0.05). (A): The heat map displays the adult HNO RSV/A/ON compared to the mock. (B) Heatmap presenting the adult HNO RSV/B/BA versus mock. (C): Heatmap for pediatric HNO RSV/A/ON in relation to mock. (D) Heatmap for pediatric HNO RSV/B/BA compared to mock.

**Supplemental Figure 5: RSV/A/ON gene expression in the ciliary subset.**

Violin plots showing per-cell expression of RSV/A/ON viral genes within the ciliated cell subset of adult (A) and pediatric (B) HNOs. Expression levels are displayed as normalized transcript counts, highlighting the distribution of viral reads across infected cells.

**Supplemental Figure 6: RSV/B/BA gene expression in the ciliary subset.**

Violin plots showing per-cell expression of RSV/B/BA viral genes within the ciliated cell subset of adult (A) and pediatric (B) HNOs. Expression levels are represented as normalized transcript counts per cell, demonstrating the presence and distribution of viral RNA within ciliated epithelial populations.

**Supplemental Figure 7: RSV genome found in pediatric basal cells and induces antiviral response in pediatric basal cells.**

(A) Hallmark Gene Set Enrichment Analysis of the basal subset in pediatric HNOs infected with RSV/A/ON**.** (B) Hallmark Gene Set Enrichment Analysis of the basal subset in pediatric HNOs infected with RSV/B/BA. The heatmap illustrates the enrichment scores and p-values for various hallmark gene sets across the different cellular subsets. The colors in the grids indicate the gene set's enrichment scores, where red signifies high enrichment and blue indicates low enrichment. (NES: Normalized Enrichment Score, adjusted p value < 0.05). (C): Dot plot of top 20 expressed genes in basal cell subset in pediatric HNOs infected with RSV/A/ON. (D) Dot plot of top 20 expressed genes in basal cell subset in pediatric HNOs infected with RSV/B/BA.

**Supplemental Figure 8: RSV genome found in pediatric ionocytes and induces antiviral response in pediatric basal cells.**

(A) Hallmark Gene Set Enrichment Analysis of the ionocytes/tuft cell subset in pediatric HNOs infected with RSV/A/ON**.** (B) Hallmark Gene Set Enrichment Analysis of the ionocytes/tuft cell subset in pediatric HNOs infected with RSV/B/BA. The heatmap illustrates the enrichment scores and p-values for various hallmark gene sets across the different cellular subsets. The colors in the grids indicate the gene set's enrichment scores, where red signifies high enrichment and blue indicates low enrichment. (NES: Normalized Enrichment Score, adjusted p value < 0.05). (C): Dot plot of top 20 expressed genes in ionocytes/tuft cell subset in pediatric HNOs infected with RSV/A/ON. (D) Dot plot of top 20 expressed genes in ionocytes/tuft cell subset in pediatric HNOs infected with RSV/B/BA.

**Supplemental Figure 9: Transcriptomic profile of basal cell subcluster in adult HNOs.**

(A) UMAP of subclustered basal cells from uninfected and RSV-infected adult HNOs. (B) Dot plots show marker genes used to define sub-basal cell clusters, with dot size representing the percentage of cells expressing each marker and color intensity (salmon to red) indicating mean expression levels. (C) Selected markers for hypothesized-RSV receptor genes in basal cell sub-clusters. (D) Hallmark Gene Set Enrichment Analysis of basal cell subset in adult HNOs infected with RSV/A/ON**.** (B) Hallmark Gene Set Enrichment Analysis of the basal cell subset in adult HNOs infected with RSV/B/BA. The heatmap illustrates the enrichment scores and p-values for various hallmark gene sets across the different cellular subsets. The colors in the grids indicate the gene set's enrichment scores, where red signifies high enrichment and blue indicates low enrichment. (NES: Normalized Enrichment Score, adjusted p value < 0.05).

**Supplemental Figure 10: RSV gene expression in the ionocytes/tuft cell subset of pediatric HNOs.**

Violin plots showing expression of RSV viral genes in ionocytes/tuft cells of pediatric HNOs. (A) RSV/A/ON genes. (B) RSV/B/BA genes. Viral RNA levels are displayed as normalized transcript counts per cell across ionocyte/tuft cell populations.

**Supplemental Figure 11: RSV gene expression in the basal cell subset of pediatric HNOs.**

Violin plots showing expression of RSV viral genes within basal cells of pediatric HNOs. (A) RSV/A/ON genes. (B) RSV/B/BA genes. Expression levels are shown as normalized per-cell transcript counts across basal populations.
