# Supplementary material for: Single cell sequencing analysis of respiratory syncytial virus–infected pediatric and adult human nose organoids reveals age differences, proliferative diversity and identifies novel cellular tropism
Source: J Infect. Author manuscript; Available in PMC 2026 Mar 22. (PMC13006048; doi:10.1016/j.jinf.2025.106617)

SUPPLEMENTAL FIGURE 1

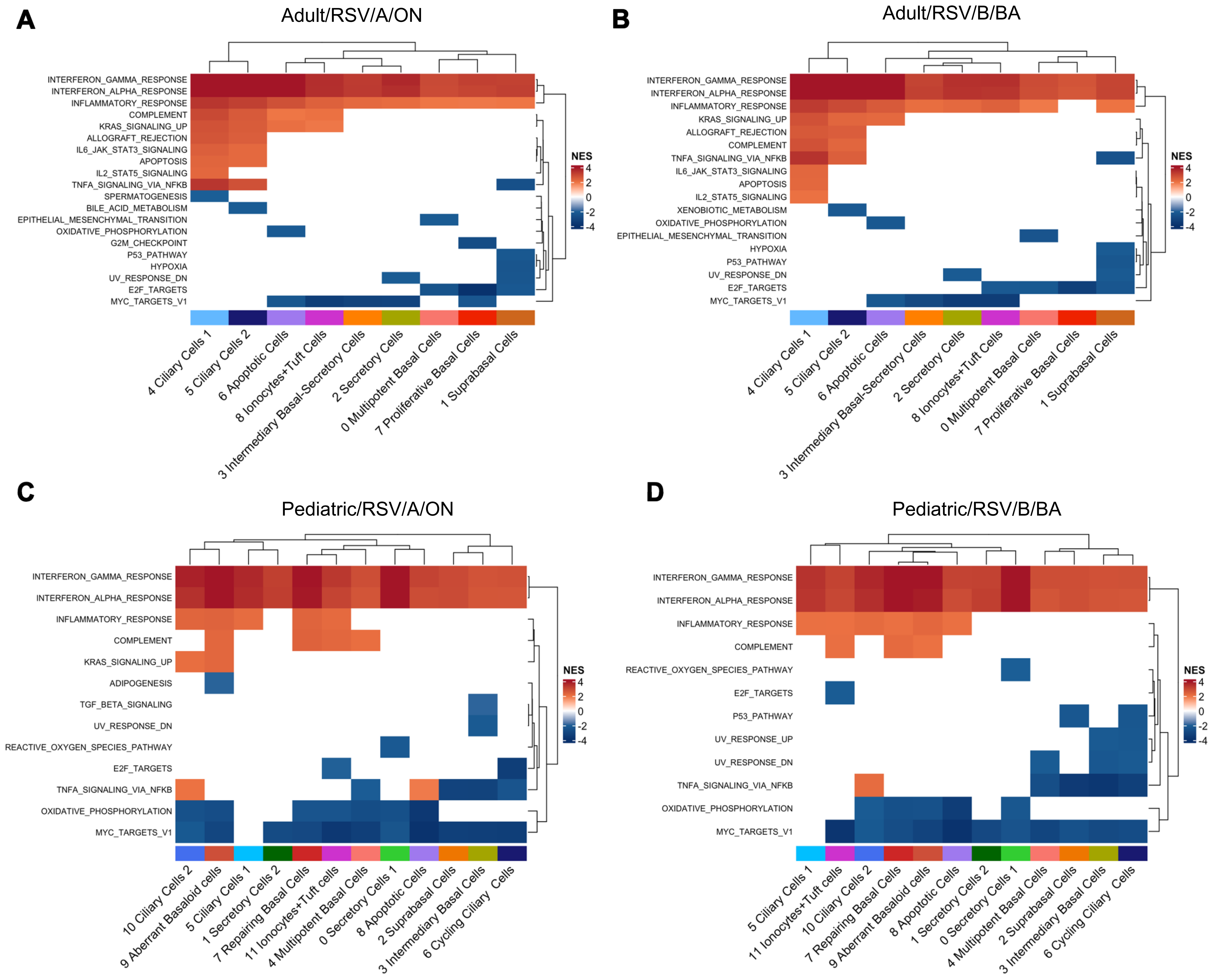

SUPPLEMENTAL FIGURE 2

A

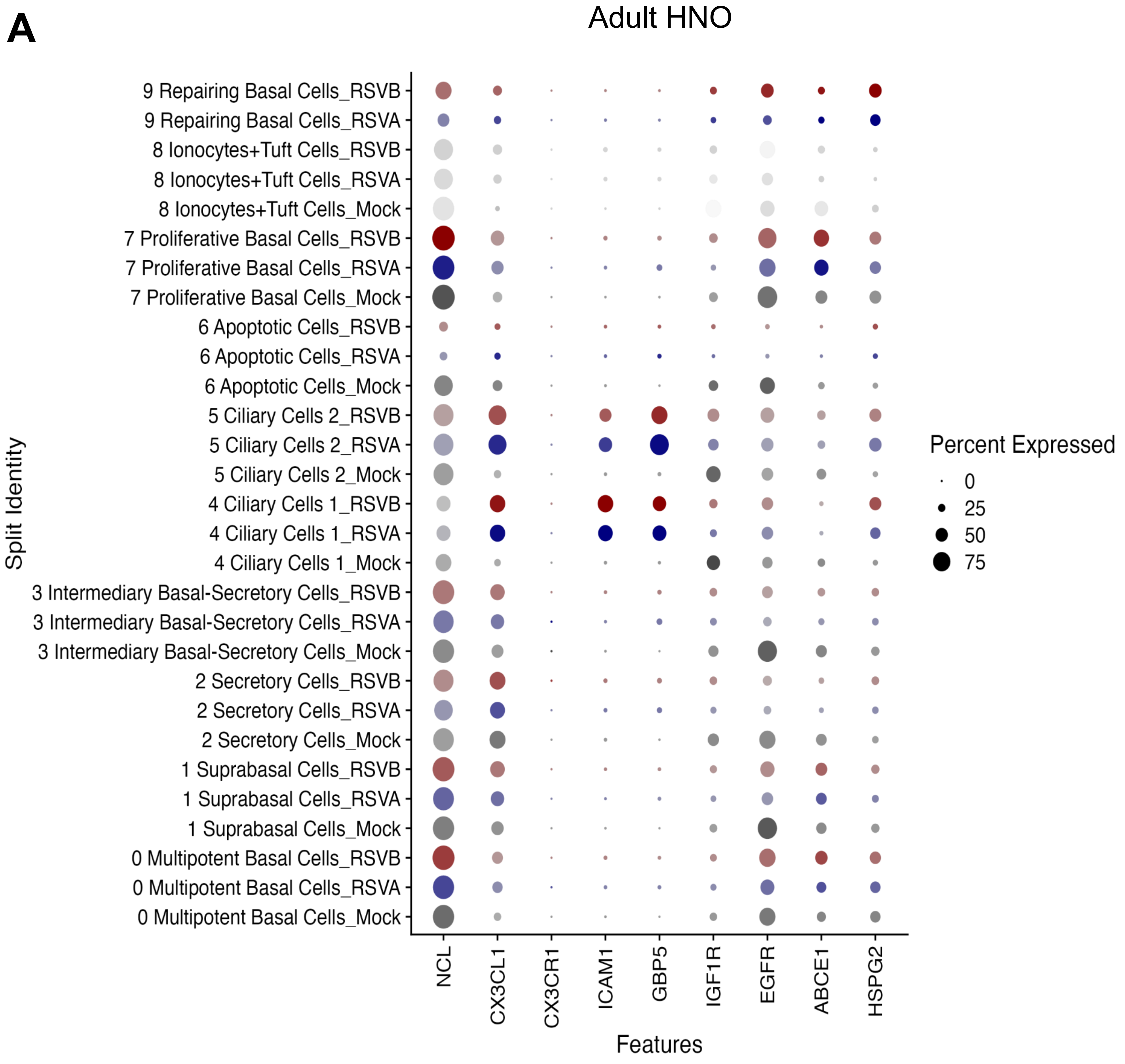

B

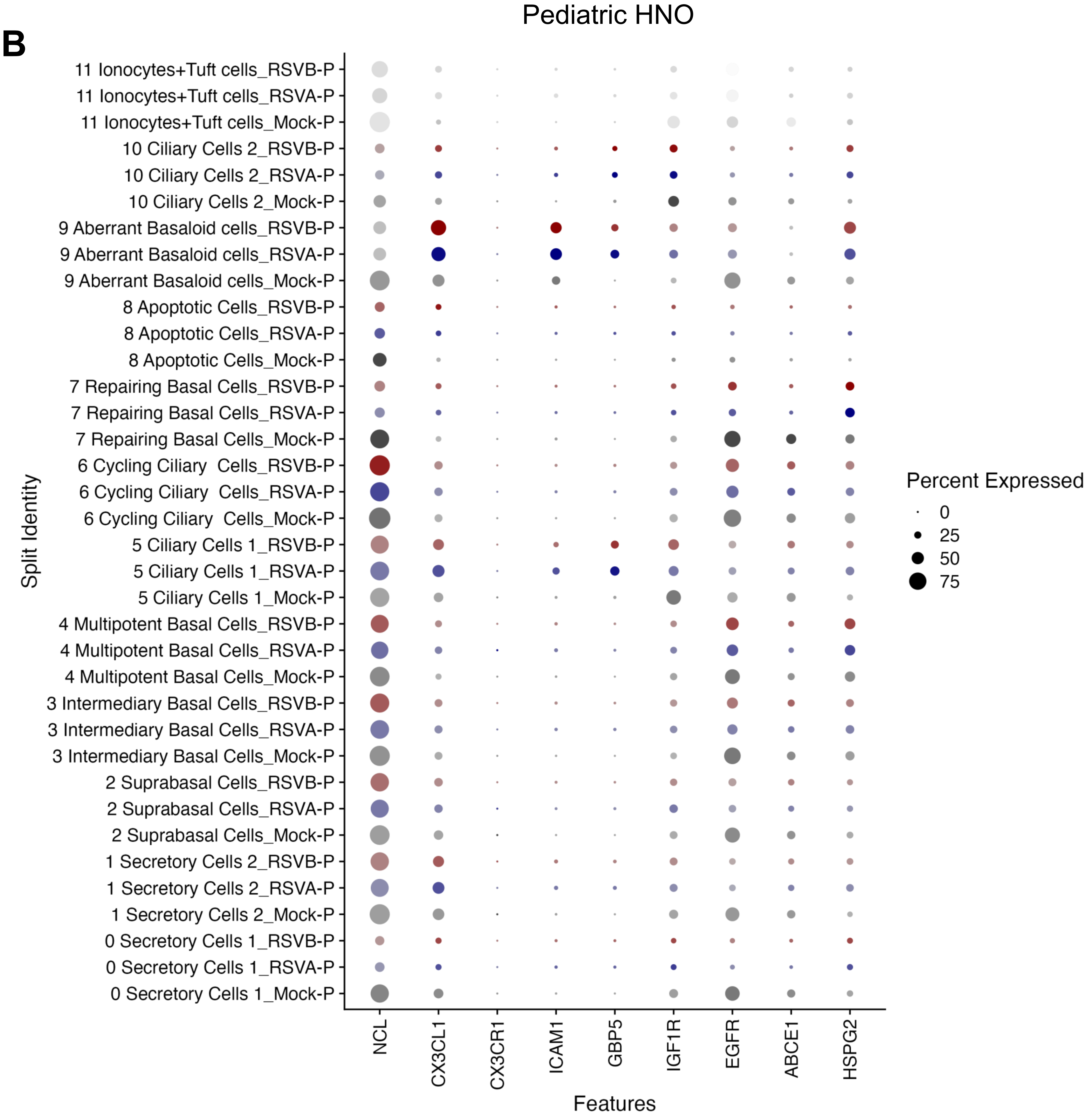

SUPPLEMENTAL FIGURE 3

A

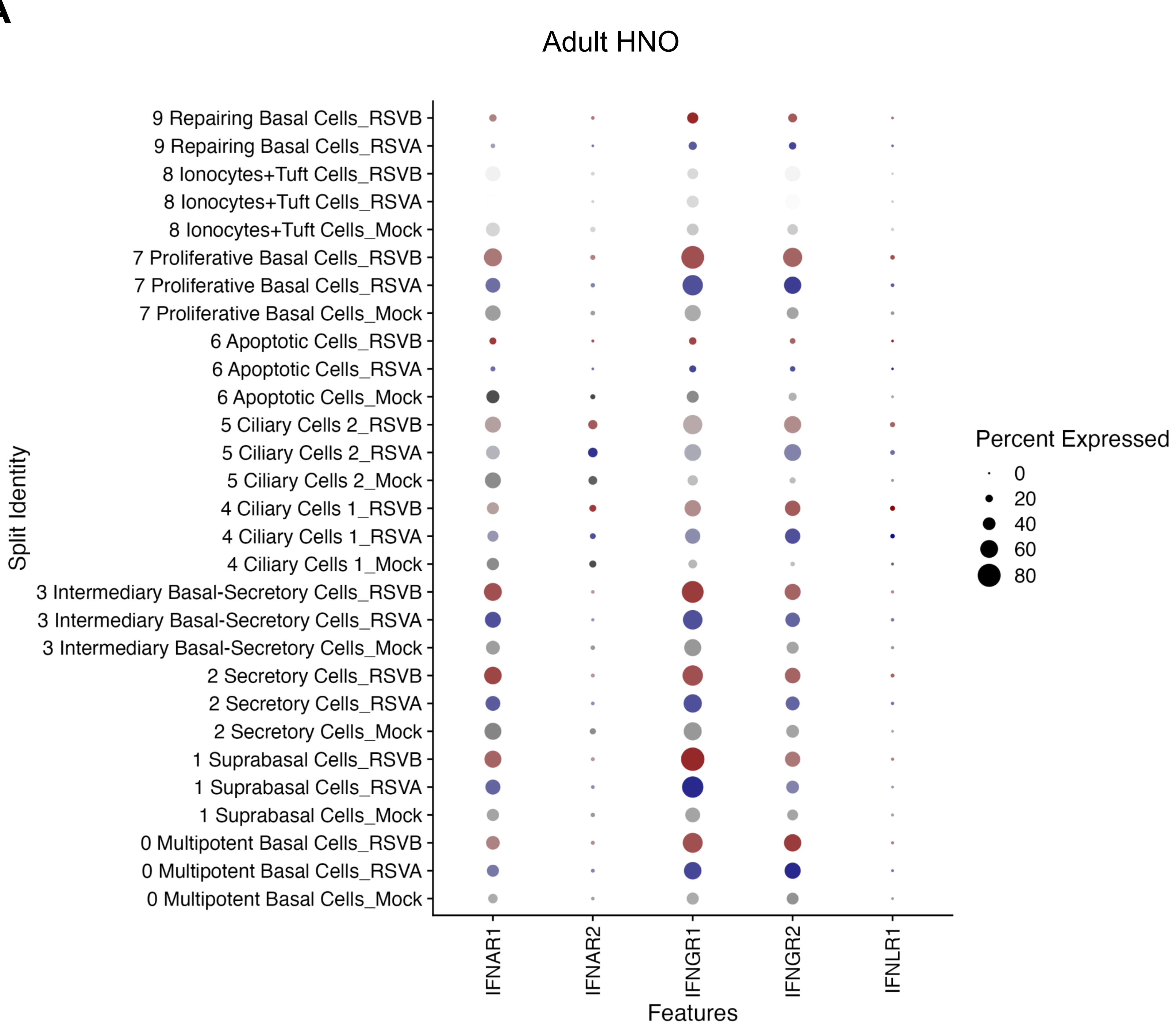

B

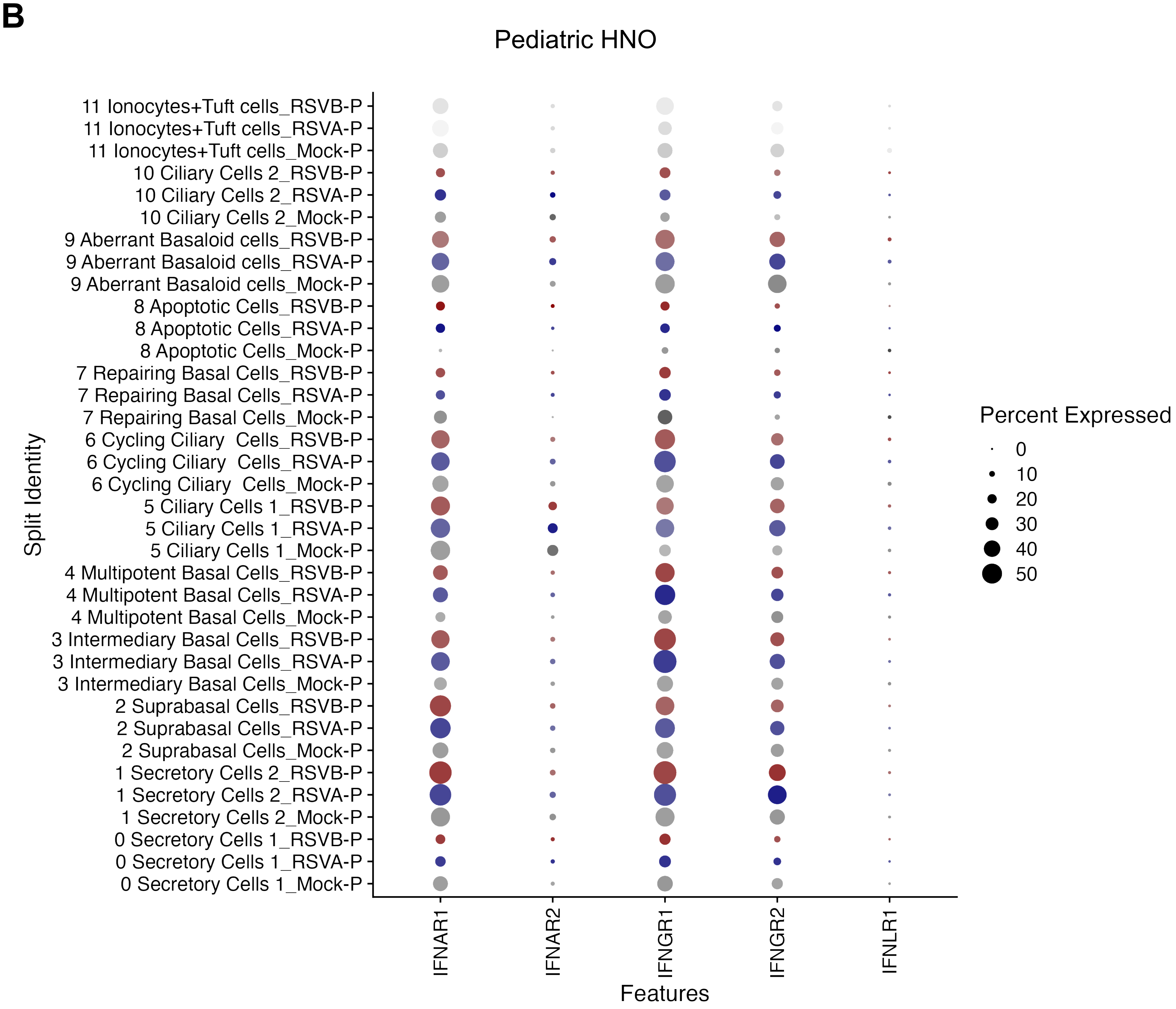

SUPPLEMENTAL FIGURE 4

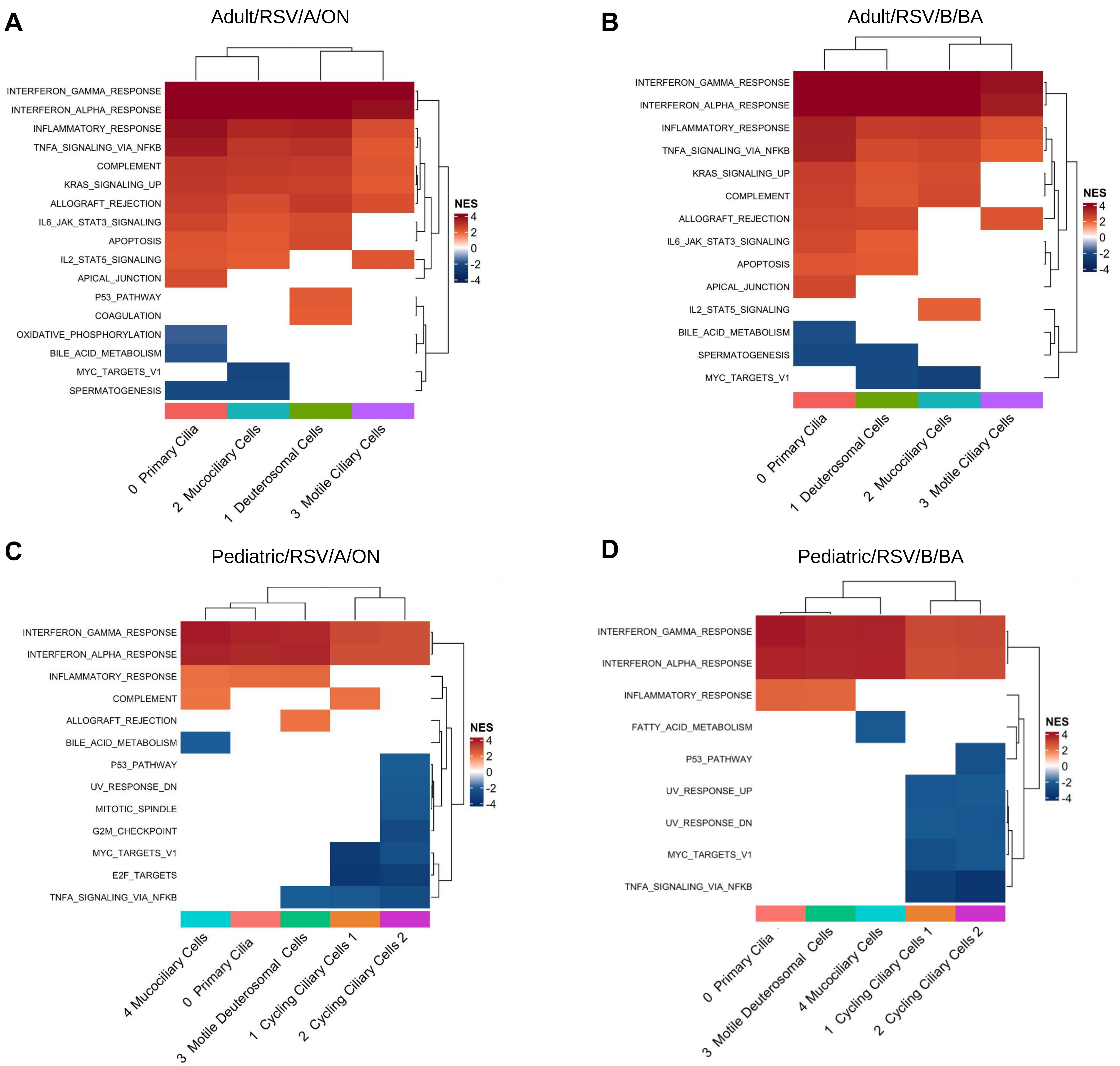

SUPPLEMENTAL FIGURE 5

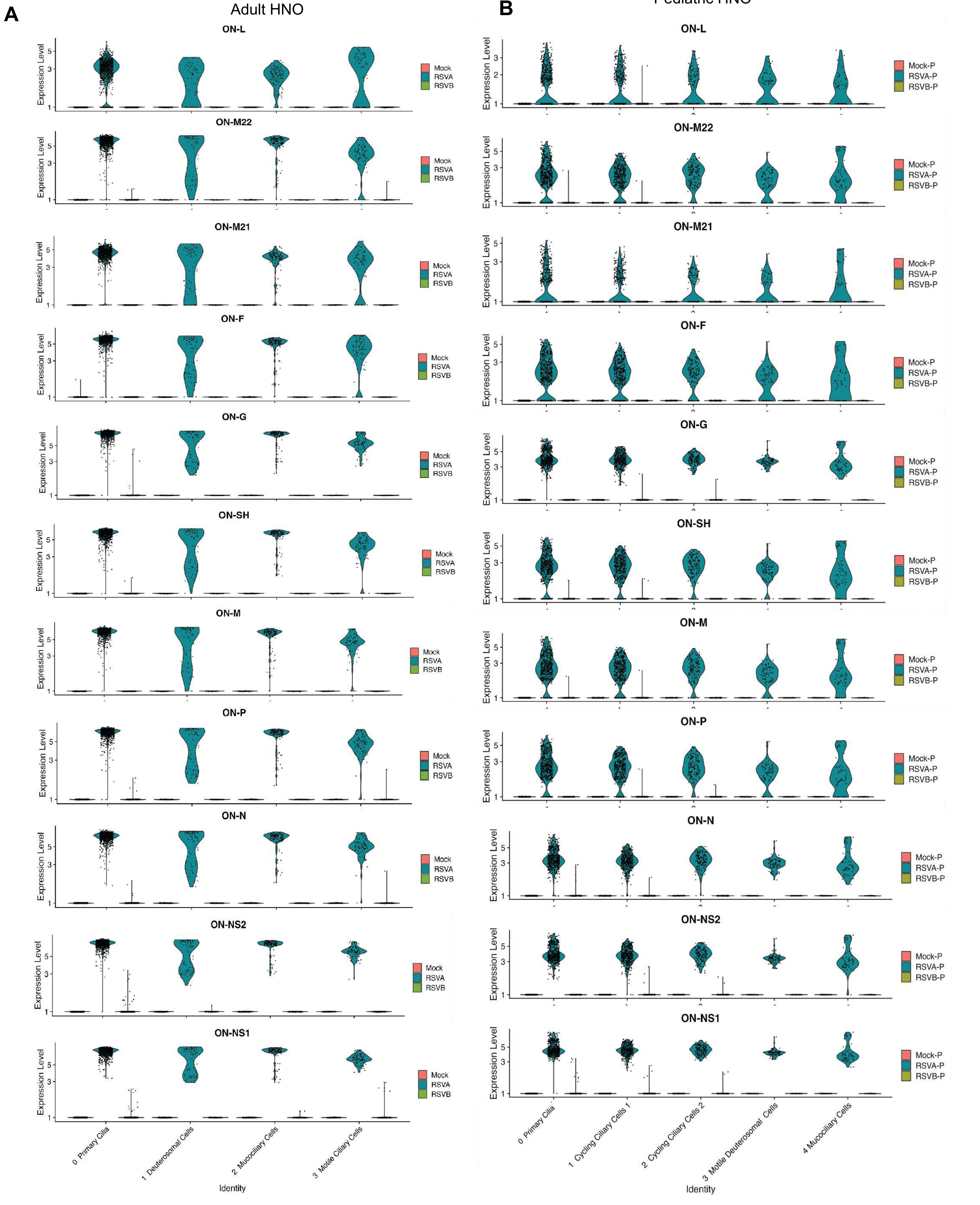

SUPPLEMENTAL FIGURE 6

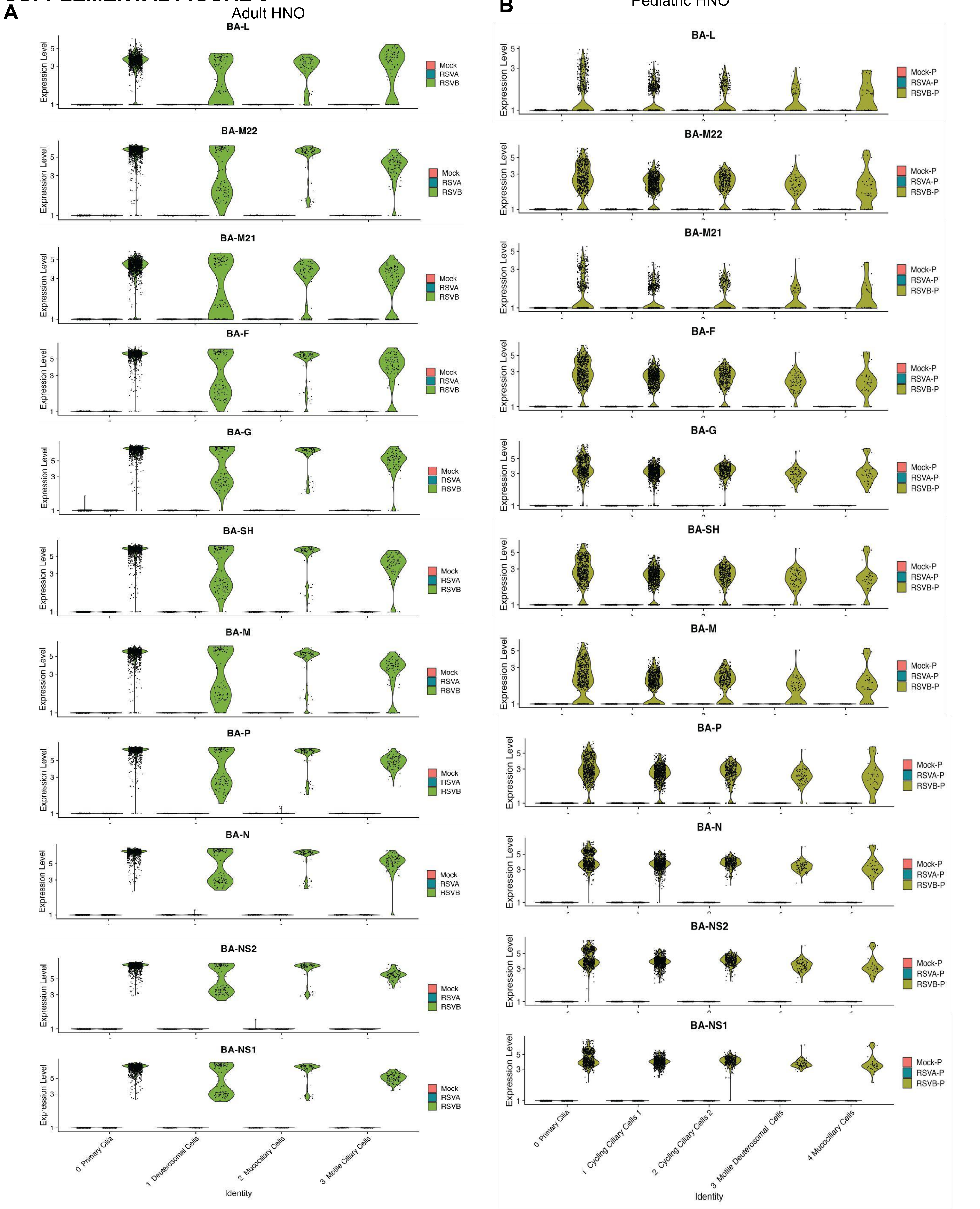

SUPPLEMENTAL FIGURE 7

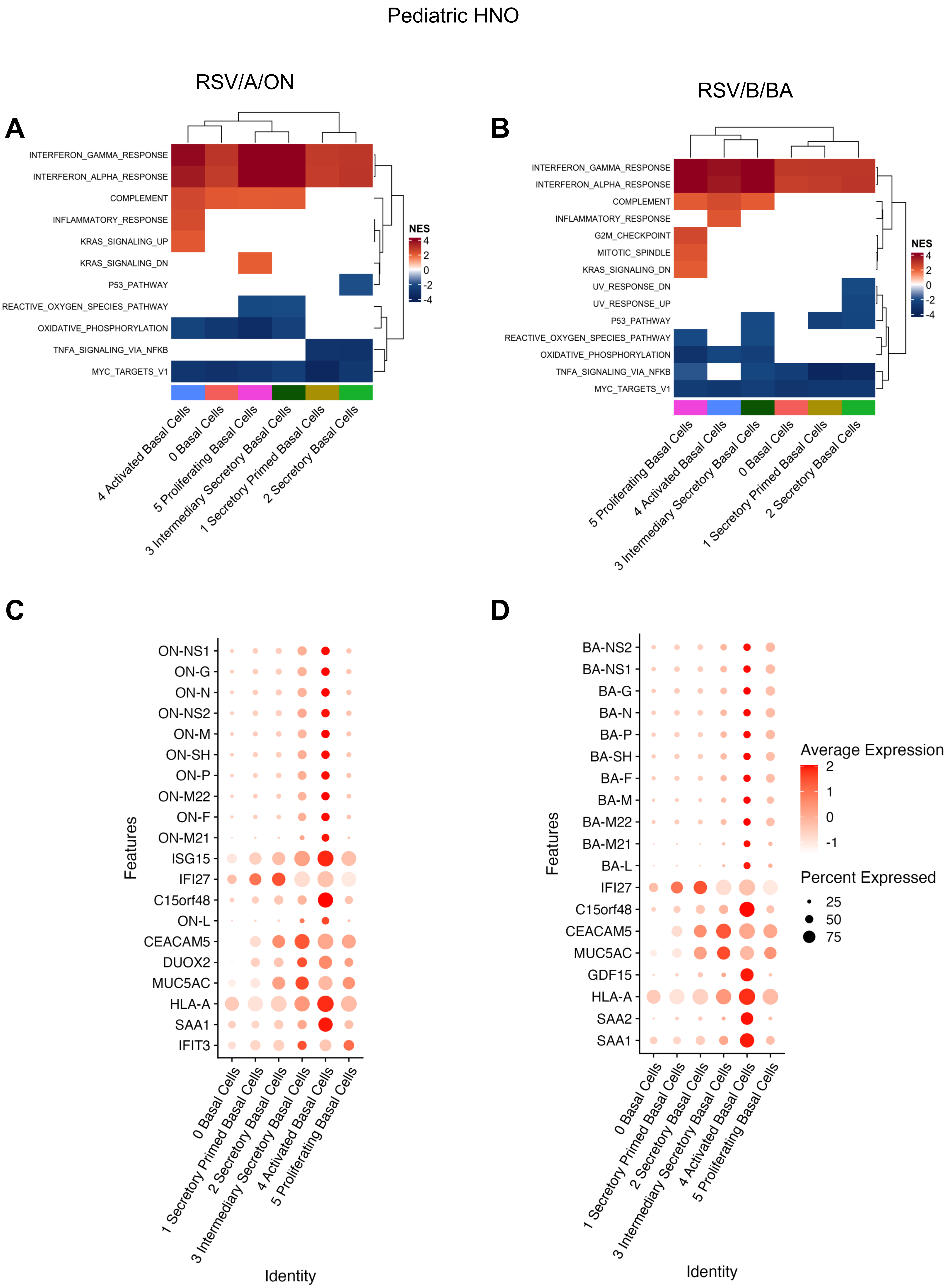

SUPPLEMENTAL FIGURE 8

Pediatric HNO

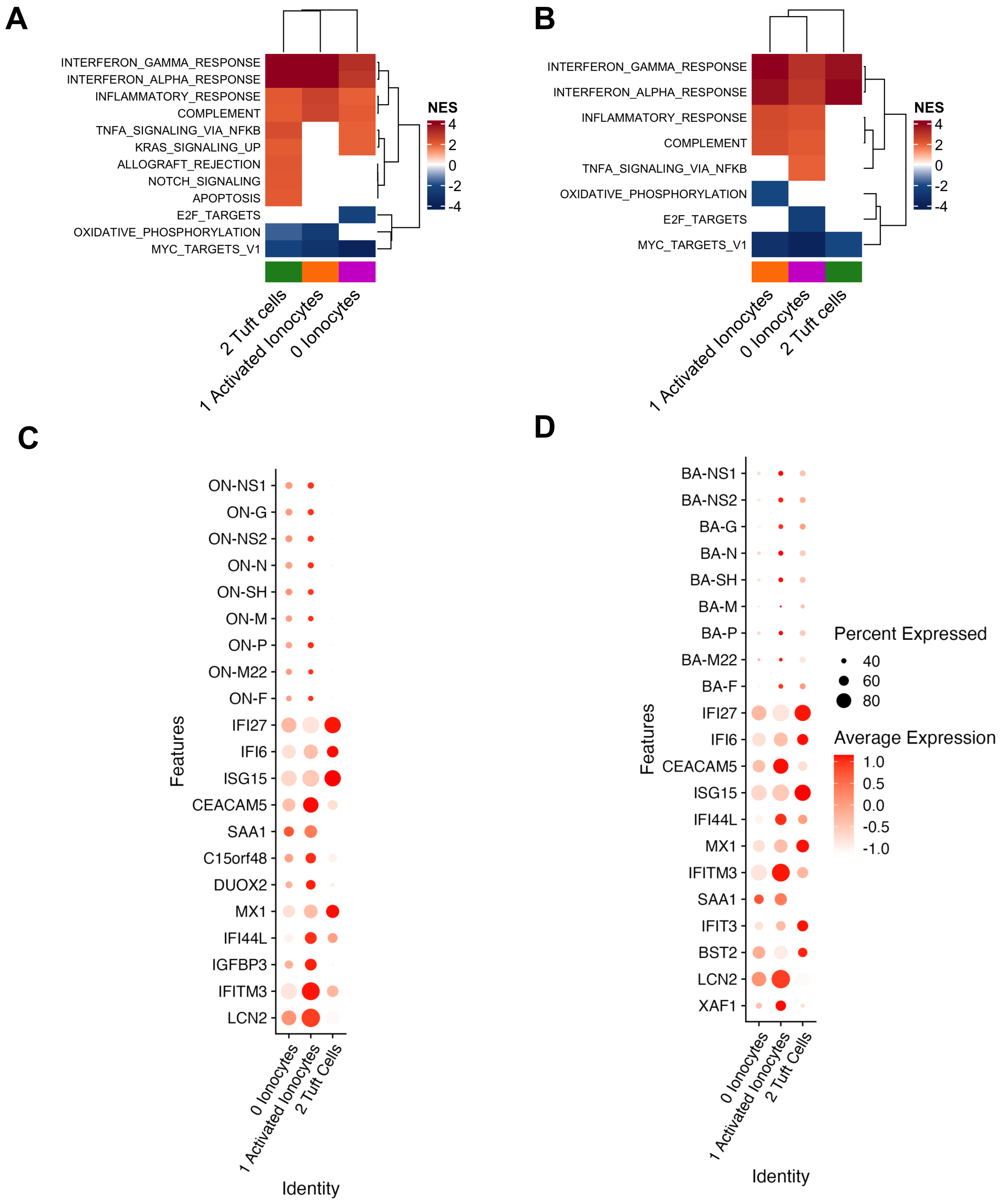

SUPPLEMENTAL FIGURE 9

Pediatric HNO

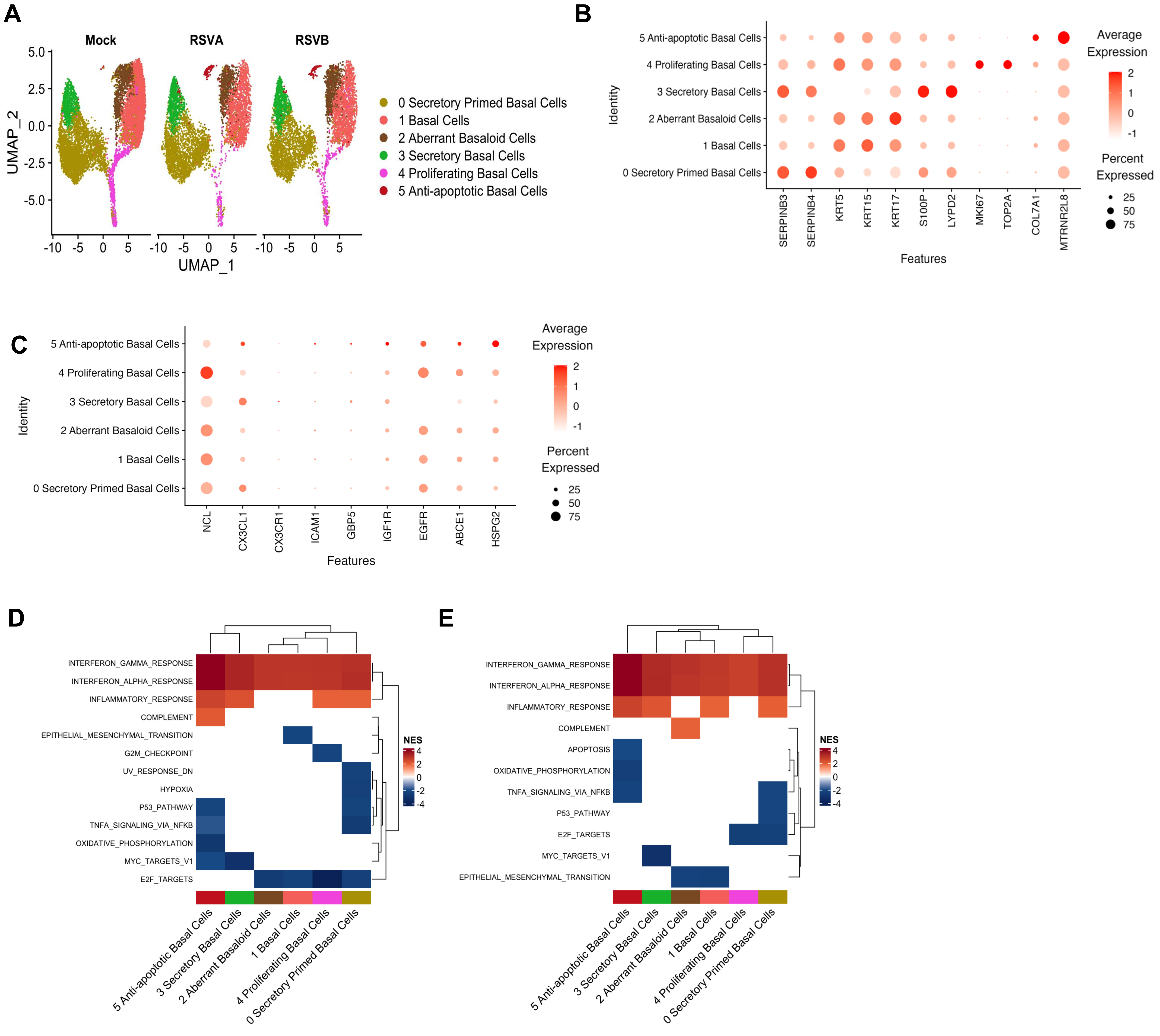

SUPPLEMENTAL FIGURE 10

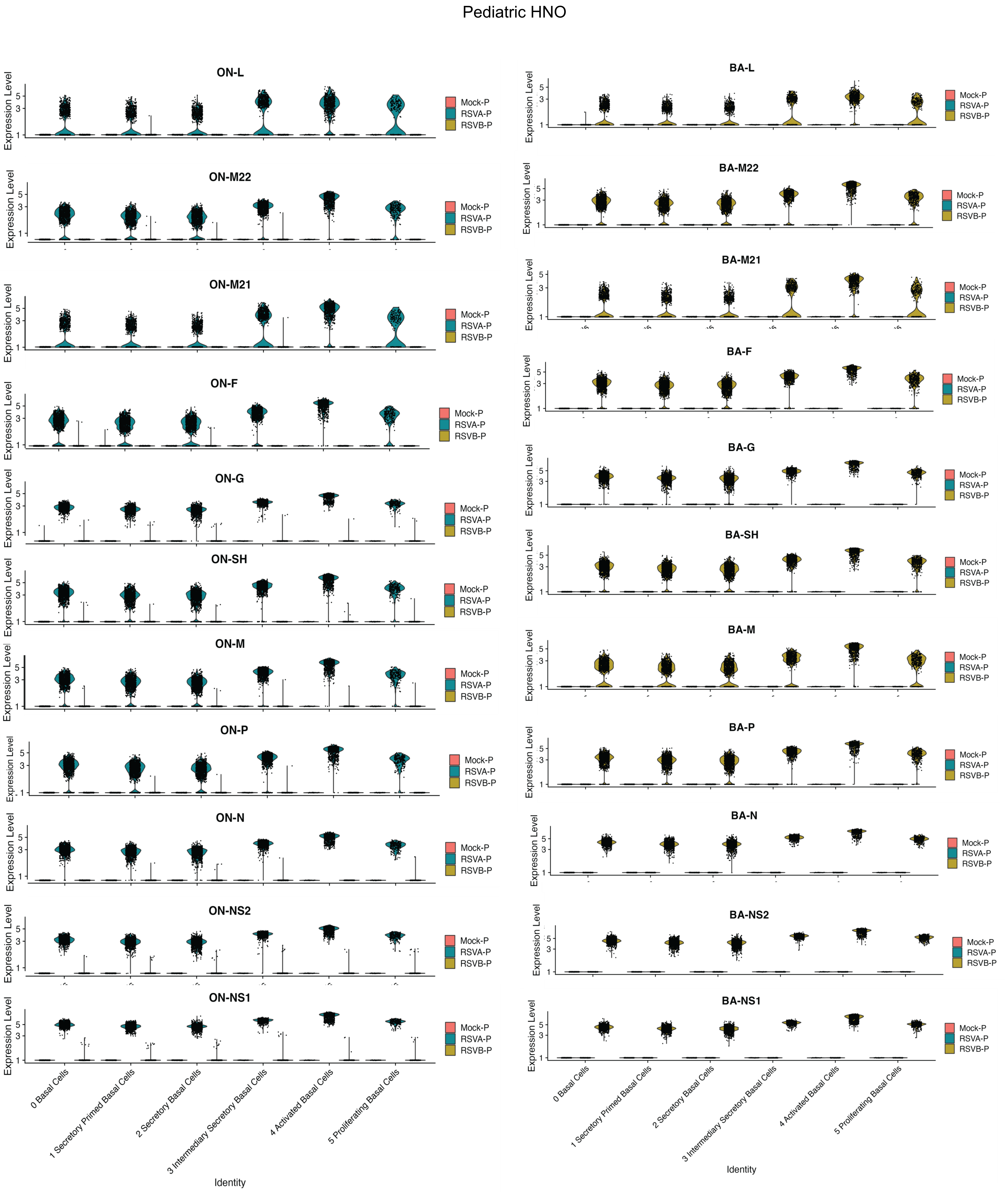

Note: The cut off for generating viral gene copies in UMAPS, was expression of 10 gene copies or higher. Whereas here in the supplemental figures we include expression levels from 1 and above.

SUPPLEMENTAL FIGURE 11

Adult HNO

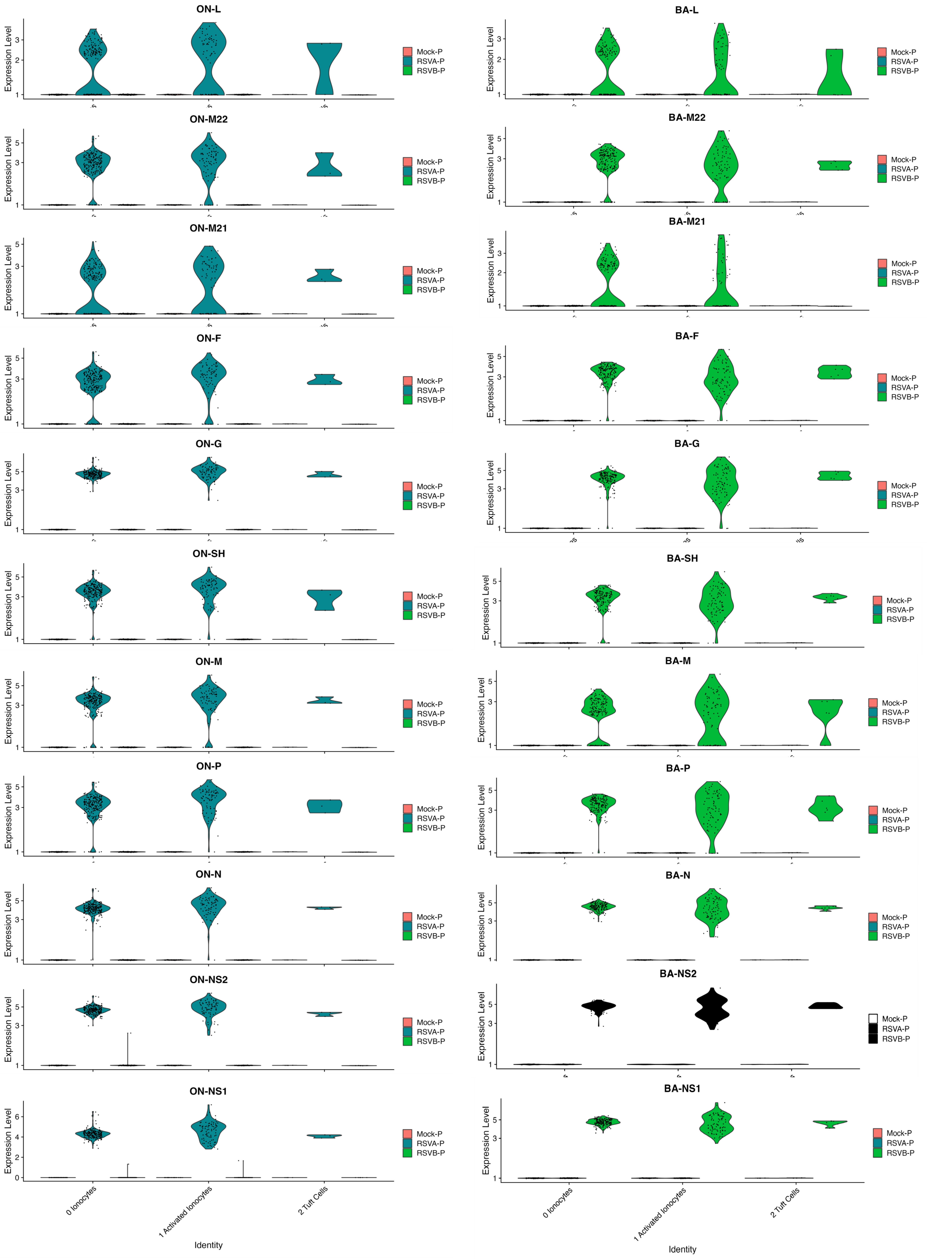

Supplement: Supplementary figures [file NIHMS2150675-supplement-Supplementary_figures.pdf]
